# The structural architecture of international industry networks in the global economy

**DOI:** 10.1371/journal.pone.0255450

**Published:** 2021-08-16

**Authors:** Thomas Sigler, Kirsten Martinus, Iacopo Iacopini, Ben Derudder, Julia Loginova

**Affiliations:** 1 School of Earth and Environmental Sciences, The University of Queensland, St Lucia, Queensland, Australia; 2 Centre for Regional Development, The University of Western Australia, Crawley, Western Australia, Australia; 3 Centre for Advanced Spatial Analysis, University College London, London, United Kingdom; 4 School of Mathematical Sciences, Queen Mary University of London, London, United Kingdom; 5 The Alan Turing Institute, The British Library, London, United Kingdom; 6 Public Governance Institute, KU Leuven, Leuven, Belgium; University of Lausanne, SWITZERLAND

## Abstract

Globalisation continuously produces novel economic relationships mediated by flows of goods, services, capital, and information between countries. The activity of multinational corporations (MNCs) has become a primary driver of globalisation, shaping these relationships through vast networks of firms and their subsidiaries. Extensive empirical research has suggested that globalisation is not a singular process, and that variation in the intensity of international economic interactions can be captured by ‘multiple globalisations’, however how this differs across industry sectors has remained unclear. This paper analyses how sectoral variation in the ‘structural architecture’ of international economic relations can be understood using a combination of social network analysis (SNA) measures based on firm-subsidiary ownership linkages. Applying an approach that combines network-level measures (Density, Clustering, Degree, Assortativity) in ways yet to be explored in the spatial networks literature, a typology of four idealised international network structures is presented to allow for comparison between sectors. All sectoral networks were found to be disassortative, indicating that international networks based on intraorganisational ties are characterised by a core-periphery structure, with professional services sectors such as Banks and Insurance being the most hierarchically differentiated. Retail sector networks, including Food & Staples Retailing, are the least clustered while the two most clustered networks—Materials and Capital Goods—have also the highest average degree, evidence of their extensive globalisations. Our findings suggest that the multiple globalisations characterising international economic interactions can be better understood through the ‘structural architecture’ of sectoral variation, which result from the advantages conferred by cross-border activity within each.

## Introduction

Network approaches to understanding global connectivity have emerged as a significant theme in political economy [1], and related approaches in economic geography. A variety of global economic processes have been conceptualised as networked structures, including global input-output systems [2, 3]; commercial and trade linkages [4, 5]; flows of capital, resources and information [6]; among others [7]. Such research has been increasingly facilitated by advancements in network science, as well as a proliferation of scholarship addressing international and inter-urban relationships from a variety of angles [8–11].

In this paper, we analyse sectoral variation in the ‘structural architecture’ of international economic networks. Our aim is to elaborate a comparative typology of international economic network structures for analysing the ‘multiple globalisations’ [12] shaped by respective industry sectors. Starting with the 666,026 intraorganisational ownership linkages connecting countries within 18,884 firm groups, we construct a series of networks that proxies the organisational differences in the ‘structural architecture’ [13] of 24 industry sectors. Using social network analysis (SNA), we begin with a descriptive metrics to understand how countries’ degree centrality by sector explains multiple core-periphery structures. This descriptive component demonstrates the great variation that exists between countries’ participation in international economic networks by sector. We then use a combination of network-oriented metrics to characterise the ‘structural architecture’ of international economic networks across each sector. By structural architecture, we mean the organisational properties underlying the way that countries are connected to one another within economic networks, in this case proxied by intraorganisational linkages representing firms’ financial organisation vis-à-vis cross-border capital flows.

This study sits among a growing number of papers using detailed datasets to study MNC networks [14–19] and identify variation and nuance within them from various perspectives [16, 20]. Our focus on the ‘architecture’ of the global economy follows a series of recent studies [21–24] applying similar network methodologies, all of which are fundamentally concerned with discerning the processes shaping economic globalisation. Globalisation, financialisation, regionalisation, uneven development (e.g. North/South, core-periphery), and (multi-) polarities are at the heart of this research [16, 23]. We build on this literature and extend it in two main ways: by combining different metrics into an overall typology of network structures, and by applying this to understand variation in the organisational properties of international economic networks by sector, eventually using a multilayer network perspective. Our analysis using countries as spatial units draws on a similar theoretical corpus of, and therefore supplements the large number of papers focussing on cities and their global connectivities [25, 26].

### Structure of international economic networks as a function of firm organisational linkages

Structural transformations in the global economy since approximately the 1970s have produced novel geographical relationships guided primarily by the activities of large MNCs [27]. The major processes underlying this shift include greater liberalisation of trade, new technologies that have enabled seamless global transportation networks and digital telecommunications, financial deregulation allowing for more complex global ownership structures, and the dissolution of trade blocs as fixtures of geopolitical relations in favour of new global and regional economic relationships [28–31]. The MNC has become the key relationship linking economies into a global system [32, 33]. This has occurred as firms source capital, labour, and other resources across international markets by establishing branch and subsidiary operations across borders with increasing ease [34]. The decidedly global geographical distribution of MNCs is thus resultant from inter-firm and intra-firm networks connecting distant spaces and places of consumption, production, and exchange [35].

Cross-cutting the evolving meaning and substance of MNC headquarters, subsidiaries, and headquarter-subsidiary relations, scholars have emphasised the importance of thinking about how places (countries, regions, cities) are structurally embedded in the global economy, particularly in relation to the increasing salience of MNCs in shaping geographical and economic relations [32, 36]. Firm-centred approaches have become pivotal to theoretical conceptualisations of economic globalisation that increasingly invoke network perspectives [10, 24, 35]. The growing application of network analysis in economic research has allowed the investigation of the structure and dynamics of global economic integration in ways that other approaches do not capture [5, 24]. This includes new ways of understanding the global positionality of places through novel relationships and communities in the structure of economic networks—many of which transcend the more obvious relations derived from earlier studies of global trade and exports [37–39].

Established ontologies that emphasise the core-periphery organisation of the global economy stem from research that shows how uneven international economic integration perpetuates inequalities [6, 37–40]. Underlying these theories, particularly those grounded in world-systems analysis [40], was the assumption that a smallish group of territorial actors (i.e. countries, and more recently city-regions) ‘commanded’ global economic flows. Though this was to some degree derived from the inherited legacy of imperial expansion and Eurocentric thinking, it also reflected the fact that the expansionary, and increasingly global, capitalist system was critically reliant on institutions, conventions, technologies, and capital from the global core. Although discursive and *ad hoc* conceptualisations of the global core prevailed, Johnston [41] and Snyder and Kick [37] were among the first to provide evidence for the core-periphery structure of the world system based on the analysis of trade networks. Successive waves of scholars have argued that industrial capacity, high added-value production, and knowledge-intensive business services continue to co-determine the systemic epicentre [40, 42, 43].

Both the hierarchical characteristics that are often argued to be inherent to corporate organisation [33, 44] and the concomitant core-periphery structures have been analysed through the lens of networks of corporate ownership relations [14, 19], input-output tables [2], and foreign direct investment [45]. We extend this literature by understanding hierarchies and global core-peripheries using corporate ownership data. The global core-periphery is understood as a function of network positionality, with countries distinguished as such within single and multi-layers resulting from the data. Our use of the term hierarchy is above all used to identify what [46] has called *hierarchical differentiation*, which refers to the ranking of elements–in this case territorial units such as countries, regions, or cities–from large to small (e.g. the rank-size rule for the distribution of cities). This differs from uses of the term hierarchy referring to the concept of *hierarchical organisation*, which carries more conceptual weight as it supposes the existence of different levels, with new properties emerging at each level (e.g. a Christallerian pattern of central places). The use of the term hierarchy in the sense of hierarchical organisation has been problematised in the study of corporate networks [47, 48], even to the point that it has been deemed “unable to adequately capture the complex nature of connectivity and spatiality developing in and between firms” ([49], p. 177). The importance of not over-interpreting the notion of hierarchy and its assumed opposite of a heterarchy [50] becomes even more pertinent (1) in light of the previously discussed impact of financialisation and digitisation on the conceptual meaning of corporate headquarters and (2) when discussing territorially aggregated patterns [51] as this would induce risks of reification and/or over-interpretation of empirical findings.

In other words, in our paper, measures of connectivity to unearth ‘hierarchies’ invoke the idea of hierarchical differentiation, thus simply acknowledging that the uneven connectivity produced within MNCs can be used to tentatively reflect on the uneven connectivity of the economies in which they are embedded [44]. Our study and others empirically confirm that corporate headquarters are concentrated in a limited number of the dense economic agglomerations in the United States, Europe and Asia [52]. In addition, numerous countries are effectively excluded from the core of the global economy, with sub-Saharan Africa, for example, having one per cent of global corporate connectivity [52].

The advent of relational perspectives and approaches to understanding the global economy has led to a revival of discussions surrounding the relevance of hierarchal differentiation within international structures, particularly as new patterns emerge of multi-layered, overlapping and regionalised nature [52]. Among these are studies that focus on the structure of global economic networks as defined by a set of industries, reflecting the complexity of industrial organisation vis-à-vis international MNC operations.

The data we use to study global economic networks reflect the ownership structures embedded in MNC activities. As Rozenblat and Pumain argue, “The architecture of ownership linkages is a marker of the decision-making and power channels between firms. Financial ownership controls the strategic orientation of the subsidiaries” ([25], p. 130). Thus given the significance of organisational network relations within MNC operations and the relative ease of proxying them [53], this paper builds on the literature using firm ownership linkages to map the structure of MNC activities and global economic networks resulting from this. Although this has come under some criticism for oversimplifying corporate structures, it is the most straightforward algorithmic structure in which the inferred connections refer to tacit information exchanges (in the broadest sense) and the existence of financial flows between locations [25, 54].

### Industry sector network structures

One body of work developing out of network-oriented studies has been concerned with the structural properties of the economic networks generated in specific sectors [2, 15, 16, 18, 52, 55, 56]. Scholars of the structure of global input-output networks report that industries are asymmetrically connected, revealing regional and global clusters of countries and the key sectors in national economies [2, 55, 56]. Countries differ in their number of industries as firms specialise in different activities and products [56] according to globally articulated divisions of labour and resource endowments. Moreover, countries are economically active in some key sectors, while less active or dormant in others [57]. Using firm data, studies similarly show that industries differ in their degree of globalisation and that position of key cities and countries within the global economy can be explained by the location strategies of MNCs specific to particular sectors [15, 16, 52].

This paper aims to demonstrate that variation in international connectivity across sectors can be captured by a network structure constructed from firm organisational linkages. Each sector’s network structure can be measured using a variety of techniques. Community detection, or the identification of groups of interacting nodes, has been used extensively to characterise the structure of corporate networks. Vitali and Battiston [18], for example, apply the Louvain method [58] to a list of 43,060 transnational corporations and reveal that the corporate ownership network demonstrates a pronounced organisation in communities where firms share similar geographical location and sector classification. Rozenblat et al. [16] identify multi-polar regionalisation in city networks when applying the spinglass clustering algorithm [59] to a global database of 1.2 million direct and indirect ownership links of three thousand MNC groups. This corroborates earlier work by Krätke [15], who analyses the corporate networks of 120 key global firms in three manufacturing subsectors (automotive industry, technology hardware and equipment and, pharmaceutical and biotechnology), revealing what Krätke and Taylor (2004) earlier called “multiple globalisations” [12]. Similarly, Wall and van der Knaap [52] examine the inter-city networks of 100 MNCs within the sectors of advanced producer services, which are purportedly proxies for larger economic processes, and all other industrial sectors. Apart from finding a strong correlation between these two industry networks, the study provides evidence of both hierarchical and heterarchical tendencies in urban corporate networks [52]. To identify nodes (cities) functioning as hubs for distinct industries, both Krätke [15] and Wall and van der Knaap [52] use measures of nodal centralities, as do several other studies focussing on international trade networks [22, 24].

Here, we focus on the sectoral variation between international networks as quantified by firm ownership ties. In many cases, these ties reflect straightforward cross-border linkages relating the economic activities of a firm and its subsidiary. Such a relationship is by definition hierarchical, in that a firm owns a subsidiary, or at least a fraction of it. Thus in quantifying ownership ties, our study proxies flows of information and capital from the subsidiary to the firm as per this assumed relationships. However, adding to the complexity of this is the fact that many MNC transactions are now digital, and linked to financial motives rather than material transactions. Thus it is very difficult to know the true nature of MNC transactions across borders, as scholars increasingly recognise the financialised dimension of international economic flows [60–62]. In other words, capital flows and therefore international economic relations are not necessarily organised according to genuine productive activity, instead often a by-product of contemporary financial markets and the ownership structures they produce.

To date, most similar studies have focussed on trade linkages [22, 24, 63]. Furthermore, few if any studies have approached international network linkages from the perspective of industry-specific ties across all sectors [15, 52, 64], which again reflects a paucity of knowledge regarding how the internationalisation strategies of firms vary across sectors, and how core-periphery structures define the role of countries within particular sectors.

Whilst network analysis has been frequently used in research to analyse industry network structures through firm data, the knowledge base has expanded as a similar set of basic network analysis measures—centrality and community analysis in particular—has been applied to more detailed and diverse databases. Although very useful and powerful as standalone network measures, they now can be used in combination with measures of global, regional and local network properties. For example, measures of assortativity, clustering coefficient and degree distributions have been successfully applied to input-output tables [2]. To our knowledge, this paper is the first to introduce a comparative methodology that combines node- and whole-of-network measures into a typology for analysing variation in the network structure of international organisational linkages across all sectors individually.

## Methods and results

### Data

Firm-level data were obtained from the Bureau van Dijk’s Osiris database [65] between mid-2016 and mid-2017. From these data, we were able to discern country locations for 18,884 firms with 666,026 subsidiaries representing 24 Global Industry Classification Standard (GICS) industry groups (based on four-digit GICS codes), which we refer to herein as sectors (to avoid confusion with firm ‘groups’). Our dataset represents 13 of the world’s major stock exchanges, including one in Africa (Johannesburg Stock Exchange), four in Asia (Bombay Stock Exchange, Shenzhen Stock Exchange, Shanghai Stock Exchange, Tokyo Stock Exchange), one in Australia (Australian Securities Exchange), three in Europe (Deutsche Börse, Euronext, London Stock Exchange), one in Latin America (Bovespa), and three in North America (Nasdaq, New York Stock Exchange, Toronto Stock Exchange). Firms were drawn from an initial list of more than 25,000 securities, ranging from 167 listed in Johannesburg to 3,756 on the Bombay Stock Exchange. The distribution of headquarters and subsidiaries as well as the ratios between them across 24 sectors is shown in Table 1.

**Table 1:** GICS industry groups (sectors) by a number of countries, headquarters and subsidiaries in the network.

Industry group (sector)	Countries	Firms	Subsidiaries	Subsidiaries/ Firm
Automobiles & Components	176	421	16,289	38.69
Banks	133	611	85,579	140.06
Capital Goods	167	2,293	74,522	32.50
Commercial & Professional Services	167	555	18,862	33.99
Consumer Durables & Apparel	108	790	19,489	24.67
Consumer Services	138	547	1,653	3.02
Diversified Financials	137	1,478	127,665	86.38
Energy	139	1,206	26,276	21.79
Food & Staples Retailing	73	175	7,279	41.59
Food, Beverage & Tobacco	160	640	1,836	2.87
Health Care Equipment & Services	109	591	2,746	4.65
Household & Personal Products	124	127	4,786	37.69
Insurance	123	170	41,682	245.19
Materials	176	3,008	55,985	18.61
Media	119	442	19,618	44.39
Pharmaceuticals, Biotechnology & Life Sciences	130	883	16,125	18.26
Real Estate	105	868	51,876	59.77
Retailing	128	635	20,817	32.78
Semiconductors & Semiconductor Equipment	67	246	4,598	18.69
Software & Services	148	1,319	25,008	18.96
Technology Hardware & Equipment	137	928	21,797	23.49
Telecommunication Services	147	148	6,275	42.34
Transportation	158	394	13,621	34.57
Utilities	109	409	1,642	4.02

As reported in Table 1, some sectors are more extensively networked across the globe than others. For example, firm networks in Materials connect 176 countries while Food & Staples Retailing links just 73 countries. Moreover, sectors differ in the depth of subsidiary networks defined by the average number of subsidiaries per headquarters in a sector (Table 1, column 5). Cross-border relations in sectors with a relatively higher number of subsidiaries per headquarters may be charaterised by activity linked to tax optimisation through mechanisms such as base erosion or profit shifting. Various financial industries (Banks, Diversified Financials, Insurance) feature a larger number of subsidiaries in relation to the number of headquarters than consumer goods and services sectors (Food & Staples Retailing, Health Care Equipment & Services).

### Network analysis specification

Though many similar studies have applied network analysis to understanding global economic connectivities between cities and other territorial scales (e.g., regions), this analysis is performed at the ‘national’ scale for multiple reasons. Conceptually, we refer to the fact that countries are conceived of as territorially bounded containers whose sovereign power supports economic activities [66, 67] in both the public and private sectors. Though this may come into question as alternate scales assume greater responsibilities vis-a-vis globalisation [68], we would maintain that countries are the dominant territorial scale at which firm, and therefore sector, activity is organised and differentiated. Firms conducting business across borders do so with very deliberate intentions and strategies that are often quite different from their domestic ones. Furthermore, there are methodological advantages to using countries, namely that each firm’s home jurisdiction is attributed to a unique ISO-2 country code, and that there is a (roughly) finite number of countries. Thus, constructing a network from ties between countries proves to be relatively straightforward, removing one of the major scalar obstacles facing those conducting research at other spatial scales.

Rooted in graph theory, network analysis looks at a system in terms of its—sometimes multi-layered—interactions among its composite units. Network analysis has been used across disciplines as a powerful tool for understanding relations from individual (node), community (subnetwork), and systemic (network) perspectives [9]. The building blocks of a network are the nodes (sometimes referred to as “vertices”), which in our case represent countries. The ties between them (sometimes referred to as “edges”) are representative of a range of overlapping relationships including financial flows, information exchange, knowledge dissemination, and upstream and downstream economic linkages [6]. Analysis of international networks by sector required MNC interorganisational ties to be transformed into country-to-country relations. Network ties were based on an International Securities Identification Number (ISIN), a unique identifier of a stock, the first two letters of which indicate the issuing country represented by their ISO 3166–1 codes, with CA for Canada, CN for China, and so on. Therefore, the relation between the Canadian headquarters and its Chinese subsidiary was recorded as a CA-CN tie.

The relationships within a given sector, say *α*, are encoded into the directed network $G^{\left[\alpha \right]}(V,E^{\left[\alpha \right]})$ composed by the set of N=|V| countries and the set of $E^{\left[\alpha \right]}=|E^{\left[\alpha \right]}|$ ties, fully represented by its adjacency matrix $A^{\left[\alpha \right]}\equiv \{a_{ij}^{\left[\alpha \right]}\}$. In this binary representation, each element $a_{ij}^{\left[\alpha \right]}=\{0,1\}$ denotes the presence (1) or absence (0) of a link from country *i* to country *j* within the sector *α*. Weighted ties are similarly encoded into a matrix $W^{\left[\alpha \right]}\equiv \{w_{ij}^{\left[\alpha \right]}\}$, where $w_{ij}^{\left[\alpha \right]}$ counts for the number of headquarters located in country *i* that own a subsidiary in country *j*.

First, we explore the variation in the properties of global industrial networks. The different relationships for sectors can be represented separately and jointly by considering each sector as a stand-alone network (one for each value of *α*) or by stacking all the sectors together in a multiplex network [69, 70]. In particular, we build a multiplex network $M={\left\{G^{\left[\alpha \right]}\right\}}_{\alpha =1,\ldots ,M}$ consisting in a collection of *M* = 24 layers, one for each GICS sector. While all layers are composed of the same nodes (countries), the connections within each layer are based on the links between countries for the respective industry sector, preserving in this way the sectoral multi-layered nature of the country-to-country interactions [71, 72].

We start by computing the in-degree and out-degree centralities for each country within each layer (sector) and compare them to the corresponding multiplex measures [73]. This simple analysis would allow us to differentiate, for example, countries (nodes) that are globally well connected in all the sectors from countries (nodes) that are hubs in a specific sector, but are much less connected to the others.

Furthermore, we complement this analysis by investigating the multilayer core-periphery organisation of the network. More specifically, we first consider the original single-layer formulation, as proposed by Ma and Mondragón [74], and apply it to each layer. This is a parameter-free method in which nodes are ranked according to a “richness” measure and then deterministically divided into two classes, namely core and periphery. Broadly speaking, the core is composed of high-degree nodes that are densely connected with one another, while nodes in the periphery are loosely connected to the core (more details on the exact procedure can be found in [74]). Secondly, we extract the multiplex core-periphery by following the generalised procedure proposed by Battiston et al. [75, 76]. In this case, the multiplex “richness” measure takes into account all the network layers at the same time. Since the number of connections between countries can vary across different layers, we properly normalise the multiplex richness measure in order to have equal contributions from all the sectors (that means setting the coefficients *c*^[*α*]^ proportional to 1/*E*^[*α*]^ (see Ref. [75] for method details).

**Fig 1:**
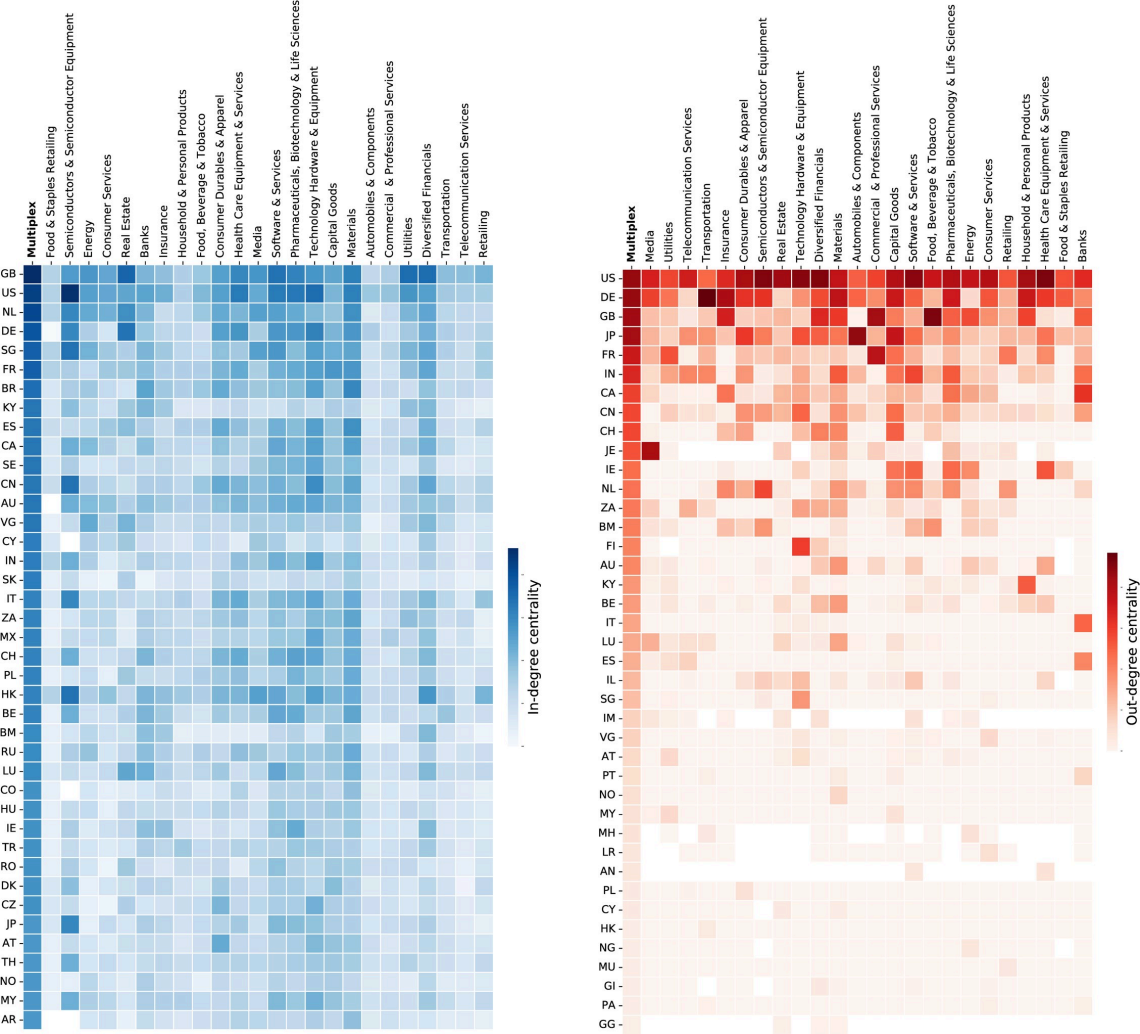
a. In-degree centralities for single-layer and multiplex industry networks. Each column corresponds to a different layer (sector) of the multiplex network, which is shown in the first column. Each row corresponds to a different country, being ordered by decreasing values of in-degree centrality according to the multiplex ranking. The darker the colour, the higher the centrality. Top 40 countries are shown by two-digit ISO-2 code. b. Out-degree centralities for single-layer and multiplex industry networks. Each column corresponds to a different layer (sector) of the multiplex network, which is shown in the first column. Each row corresponds to a different country, being ordered by decreasing values of out-degree centrality according to the multiplex ranking. The darker the colour, the higher the centrality. Top 40 countries are shown by two-digit ISO-2 code.

The variation in the connectivity patterns across sectors between the single-layer and multi-layer perspectives is depicted in Fig 1A and 1B. Specifically, Fig 1A identifies to what extent countries are involved in specific sectors based on in-degree centrality (the number of outward ties a country has from international headquarters to domestic subsidiaries). Fig 1B shows the relative importance of countries across sectors based on out-degree centrality (the number of outward ties from headquarters in a country to subsidiaries overseas). Countries (lines) in both heatmaps are ranked according to the values in the first column, which represents the multiplex.

**Fig 2:**
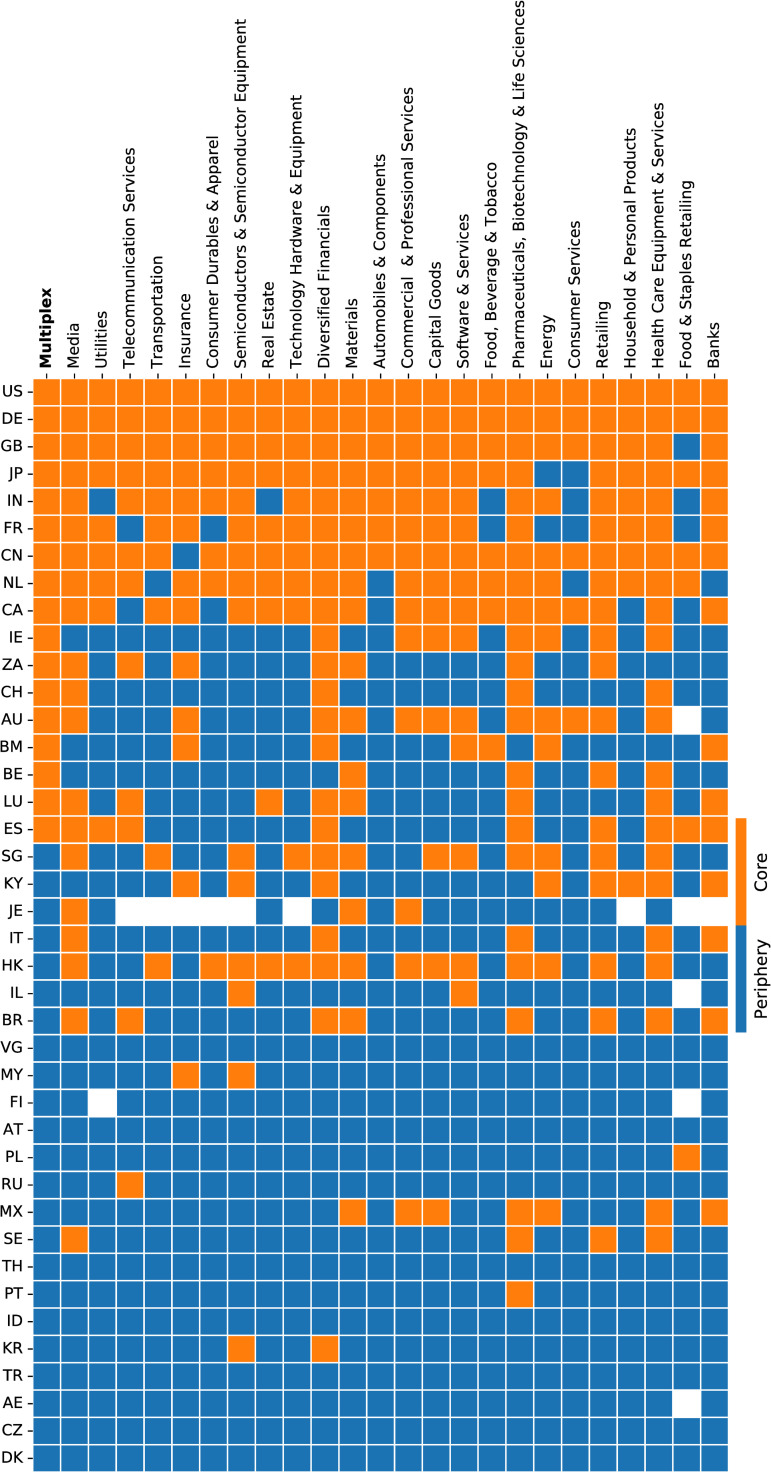
Core-periphery organisation of global corporate networks across sectors. Each column corresponds to a different layer (sector) of the multiplex network, which is shown in the first column. Each row corresponds to a different country, with associated cells coloured in orange or blue according to its position within the core or the periphery in the considered network (column). Rows are ordered by decreasing values of multiplex richness [75]. Therefore, the ordering is induced by the multiplex core-periphery organisation (first column). Top 40 countries are shown by two-digit ISO-2 code.

Fig 2 provides graphical evidence that each network is defined by a limited number of “core” countries active in some sectors, while less active or absent in others. Rows are ranked according to a multiplex richness measure that classifies countries into core (orange cells) and periphery (blue cells). As before, the ordering of countries is induced by the ranking of the first column that follows the multiplex core-periphery organisation. The remaining columns show the core-periphery structure layer by layer.

We highlight three revealing patterns in the properties of the sector networks. First, some countries are in the network “core” in both the multiplex and the single-layer representation. The global “core” countries—the United States, Germany, the United Kingdom, Japan, India, France, China, the Netherlands, Canada—feature across most sectors, and are at the top of the multiplex-core. Second, other countries, such as Ireland, South Africa, Switzerland, Australia, Bermuda, Belgium, Luxembourg and Spain, are in the core only when the multi-layered nature of the system is considered. They are in the periphery for most other sectors. Offshore financial centres (e.g. Bermuda, Singapore, the Cayman Islands, Jersey, the British Virgin Islands) feature prominently within particular networks [77], indicating that there are favoured corporate structures vis-à-vis industries and tax havens. Thirdly, countries with specialised export economies (e.g. Italy, Israel, Brazil, Malaysia, Poland, Russia, Mexico, Sweden, and South Korea) form a core of some sectors, but not others.

In order to identify a typology of network structures based on firm organisational linkage data, we use and combine four metrics at node and network levels to characterise variation across sector networks. We consider the following metrics as indicative of different facet of network structure as follows:

- **Network density***D*^[*α*]^ is measured as the proportion of existing ties in a layer *α* relative to the total number possible. The calculation of *D* returns a value in a range between 1 (a maximally connected graph) and 0 (a graph without ties). Higher values of *D* suggest the presence of dense webs of interconnections between countries. When applied to industry networks, higher *D* indicates a relatively more inter-connected sector.
- The **Average Clustering Coefficient** ⟨*C*^[*α*]^⟩ is a measure of the degree to which nodes in a layer *α* tend to cluster together in terms of triads. When applied to a single node, *C* is a measure of how complete the neighbourhood of a node is, i.e., how much the neighbours of a node are neighbours themselves. ⟨*C*^[*α*]^⟩ is then the average value over all of the nodes in the layer. In terms of the industry networks, we interpret this to indicate the degree to which sectors are organised as sub-networks. Though this often conforms to regions of the world (e.g. Latin American firms connecting through regional subsidiaries), it may also be indicative of a fragmented structure as determined by non-spatial proximities [78, 79].
- **Average Degree** ⟨*k*^[*α*]^⟩, representing the average number of connections of the nodes. Larger values of ⟨*k*⟩ in industry networks point to more densely connected networks, revealing sectors that are more globalised.
- Finally, the **Assortativity Coefficient** *r*^[*α*]^, defined as the Pearson’s correlation coefficient of degree between pairs of nodes [80], gives a compact measure of node-node degree similarity within each layer *α*. Negative values of *r*^[*α*]^ (disassortativity) of the industry networks highlight the tendency for an sector to have highly connected countries linked to poorly connected countries and vice versa. Contrarily, positive values are found when countries are mostly connected to countries of the same degree class. Larger values of negative *r* are interpreted as industries that exhibit strong international hierarchical differentiation [81, 82] and therefore as an indicator of a core-periphery network organisation.

After the computation of each, these measures were used to compare the differentiated network structures against idealised network typologies. The combination of the four network metrics indicates how well-connected each sector graph is, and accordingly the level of globalisation of the entire network as well as the individual countries within it. In this analysis, the multiplex nature of our data set is not considered. Therefore, each metric is independently calculated for each layer *α*.

### Idealised network typologies

**Fig 3:**
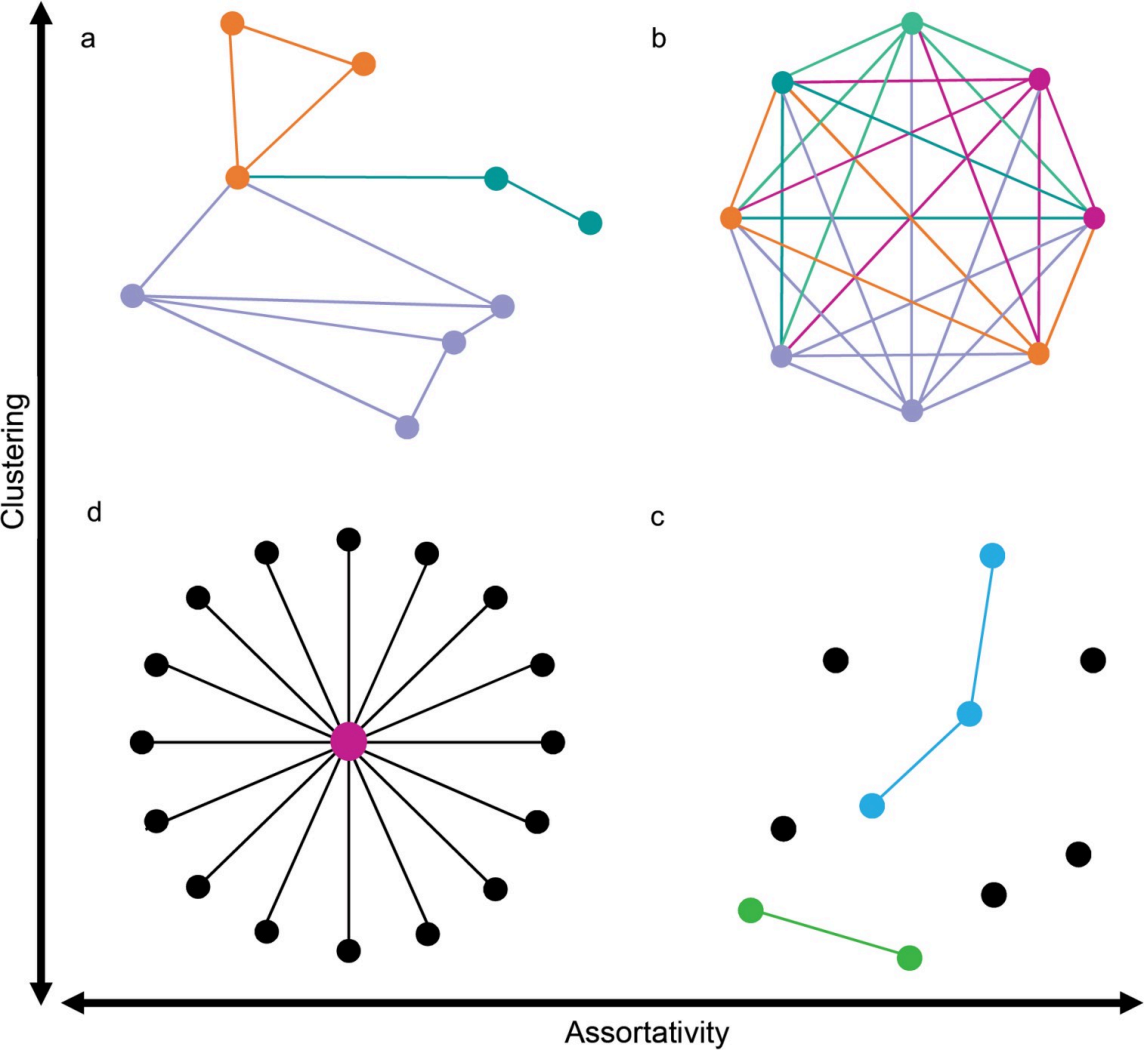
Idealised graph typology to explain network structures. a–polycephalous, b–clique-like, c–segmented-decentralised, d–star-like.

Different classes of definitions have been developed to characterise the network structures in various fields such as complex networks, sociology, biology and economics [83–85]. The typology of four models in Fig 3 provides a way of categorising international economic networks by sector. The first two types (star-like and clique-like) include the networks with hierarchically and heterarchically differentiated structures and are common in corporate and other types of networks [52, 82]. The polycephalous and segmented-decentralised types have been previously identified in research on networked social movements [86–89] and represent networks with less regular patterns of connections. Irregular network structures are relevant to our typology as industry networks are likely to reflect regional rather than global character, with autonomous communities that are adjacent through intermediaries [16, 90]. The four models are described as follows:

- A **Star-like** network type (Fig 3D) has a relatively large number of connections to a single node, or a very small number of central nodes. This results in networks that are centralised around a small core, but where neither nodes nor subnetworks are well-connected.
- A **Clique-like** network type (Fig 3B) represents a densely connected network where all nodes are adjacent to one other, creating tightly connected groups characterised by a relatively high clustering and lower disassortativity.
- A **Polycephalous** network type (Fig 3A) occurs when a small number of core nodes connect sub-networks, or communities. This is characterised by higher disassortativity than clique-likes, and polycephalous networks exhibit a higher clustering than stars.
- Finally, a **Segmented-Decentralised** type (Fig 3C) characterises networks composed of semi-autonomous communities and nodes (isolates). Sectors with a segmented-decentralised structure are disassortative (but more assortative than stars) and diffused rather than clustered.

### Comparative analysis of international economic network structures

In Table 2, we provide results for the assortativity coefficient (r), average degree (⟨k⟩), average clustering coefficient (⟨C⟩), and density (D). Together, they report variation in the network properties of each sector.

**Table 2:** Network properties of 24 GICS sector networks.

Industry group	Density (*D*)	Average clustering coefficient (⟨*C*⟩)	Average degree (⟨k⟩)	Assortativity coefficient (*r*)
Automobiles & Components	0.029	0.507	5.148	-0.642
Banks	0.064	0.613	8.481	-0.718
Capital Goods	0.069	0.784	11.425	-0.695
Commercial & Professional Services	0.040	0.747	6.587	-0.670
Consumer Durables & Apparel	0.071	0.631	7.556	-0.610
Consumer Services	0.043	0.532	5.942	-0.517
Diversified Financials	0.066	0.650	8.993	-0.562
Energy	0.050	0.521	6.835	-0.514
Food & Staples Retailing	0.041	0.199	2.959	-0.725
Food, Beverage & Tobacco	0.045	0.677	7.125	-0.536
Health Care Equipment & Services	0.069	0.689	7.395	-0.613
Household & Personal Products	0.054	0.708	6.645	-0.696
Insurance	0.057	0.701	6.911	-0.724
Materials	0.069	0.785	12.068	-0.627
Media	0.062	0.622	7.277	-0.579
Pharmaceuticals, Biotechnology & Life Sciences	0.073	0.721	9.431	-0.690
Real Estate	0.055	0.413	5.695	-0.415
Retailing	0.038	0.421	4.828	-0.622
Semiconductors & Semiconductor Equipment	0.105	0.678	6.896	-0.634
Software & Services	0.065	0.645	9.541	-0.571
Technology Hardware & Equipment	0.071	0.700	9.606	-0.577
Telecommunication Services	0.030	0.434	4.381	-0.604
Transportation	0.039	0.641	6.076	-0.531
Utilities	0.052	0.502	5.651	-0.556
**Median**	**0.056**	**0.643**	**6.904**	**-0.612**

**Fig 4:**
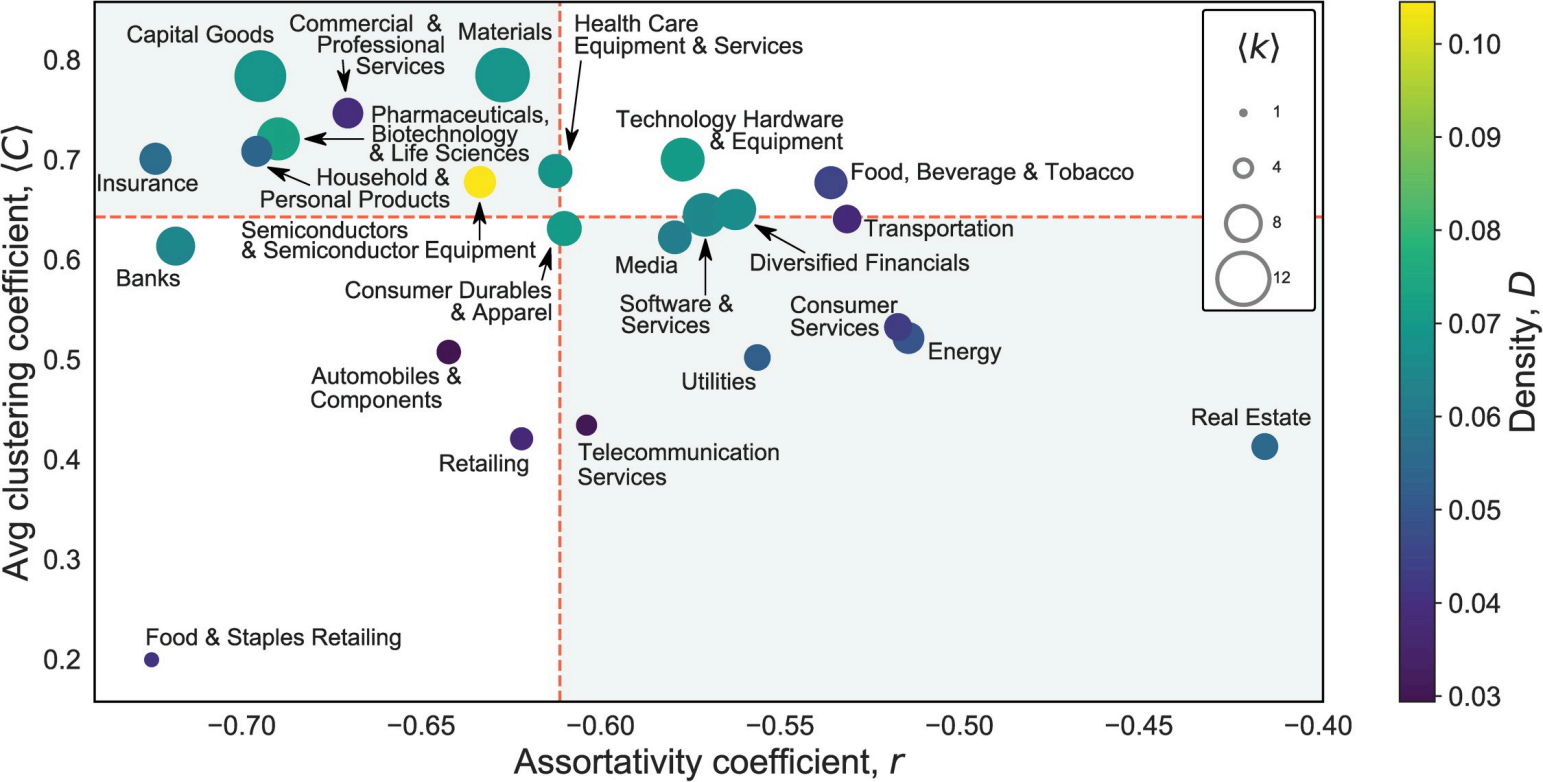
Comparison of D, ⟨*C*⟩, r, and ⟨*k*⟩ of 24 GICS sector networks. Central horizontal and vertical axes represent median values of *C* and ⟨*r*⟩ across sectors (0.64 and -0.61, respectively).

The four different network characteristics are plotted in Fig 4, and explained in Table 3 below.

**Table 3:** Properties of four idealised network types in relation to median values.

Idealised Network Type	Average Clustering Coefficient	Assortativity	Industry Groups
Star-Like	Lower	Lower	Automobiles & Components
			Banks
			Food & Staples Retailing
			Retailing
Clique-Like	Higher	Higher	Diversified Financials
			Food, Beverage & Tobacco
			Software & Services
			Technology Hardware & Equipment
Polycephalous	Higher	Lower	Capital Goods
			Commercial & Professional Services
			Health Care Equipment & Services
			Household & Personal Products
			Insurance
			Materials
			Pharmaceuticals, Biotechnology & Life Sciences
			Semiconductors & Semiconductor Equipment
Segmented-Decentralised	Lower	Higher	Consumer Services
			Consumer Durables & Apparel
			Energy
			Media
			Real Estate
			Telecommunication Services
			Transportation
			Utilities

Fig 4 shows the position of each sector network, with average clustering coefficient ⟨*C*⟩ and assortativity coefficient *r* on respective axes, and the colour representing density *D* and the size reflecting average degree ⟨*k*⟩. Each of the four quadrants relatively defined by the means of assortativity and average clustering coefficient reflects a combination of the network characteristics.

Specifically, the top-right quadrant of the plot in Fig 4 contains industry networks with ⟨*C*⟩ and *r* higher than the medians (0.64 and -0.61, respectively), indicating a tendency to the idealised clique-like type of network structure. The sectors in this group are well connected across the world (the average degree ⟨*k*⟩ ranges between 7.13 and 9.61, and density *D* ranges between 0.05 and 0.07), including Technology Hardware & Equipment; Food, Beverage & Tobacco; Diversified Financials. Higher clustering and relatively higher assortativity in these networks are linked to less hierarchically differentiated organisation and denser global connectivity.

The top-left quadrant in Fig 4 consists of sectors with ⟨*C*⟩ higher and *r* lower than the medians, indicating a polycephalous type. The sectors in this group are characterised as high-technology sectors (e.g., Pharmaceuticals, Biotechnology & Life Sciences), but very diverse, with network density *D* ranging from 0.04 (Commercial & Professional Services) to 0.11 (Semiconductors & Semiconductor Equipment), and average degree ⟨*k*⟩ ranging from 6.65 (Household & Personal Products) to above 11 (Capital Goods and Materials). Sectors in this group are generally clustered but more highly disassortative, indicating a tendency for a small number of countries to emerge as highly but more selectively connected hub nodes.

The bottom-left quadrant in Fig 4 features ⟨*C*⟩ and *r* both below the medians and represents the idealised star-like type. This group includes relatively few sectors, characterised by strong hierarchical differentiation that is centralised around one key node or a small, centralised clique. This includes Automobiles & Components, Retailing, Food & Staples Retailing, which also exhibit very low network density (*D* less than 0.41) and average degree (⟨*k*⟩ less than 5.20) indicative of less extensively connected global networks.

Finally, the bottom-right quadrant in Fig 4 includes industry networks with *r* higher than the median but ⟨*C*⟩ lower than the median. These networks are segmented-decentralised and include sectors with no clear core and with less extensive global connections. Network density *D* and average degree ⟨*k*⟩ are rather low and many of the sectors are state-related (Utilities; Telecommunication Services; Energy) or highly localised (Real Estate, Consumer Services, Media) sectors.

Comparing the network properties, a striking pattern is that all sectors display a negative assortativity coefficient, indicating that nodes with a higher degree tend to be most connected to nodes with a lower degree, and vice versa. One explanation of the negative assortativity is that lower-degree countries such as Costa Rica and Cameroon are connected mostly to higher-degree countries such as the United States and France rather than one another, indicating a core-periphery structure of corporate connections. Negative assortativity has also been found in the global input-output network [2] as high-degree sectors (e.g. construction) take inputs from low-degree sectors (e.g. transport services). From a network theory perspective, this may be framed as ‘preferential attachment’ where more connected nodes (countries) are more likely to receive new links [91–93]. Disassortativity in the network is stronger in sectors where a small number of countries are home to MNCs with an extensive global footprint—for example, Banks (-0.72), Food & Staples Retailing (-0.73), and Insurance (-0.72).

**Fig 5:**
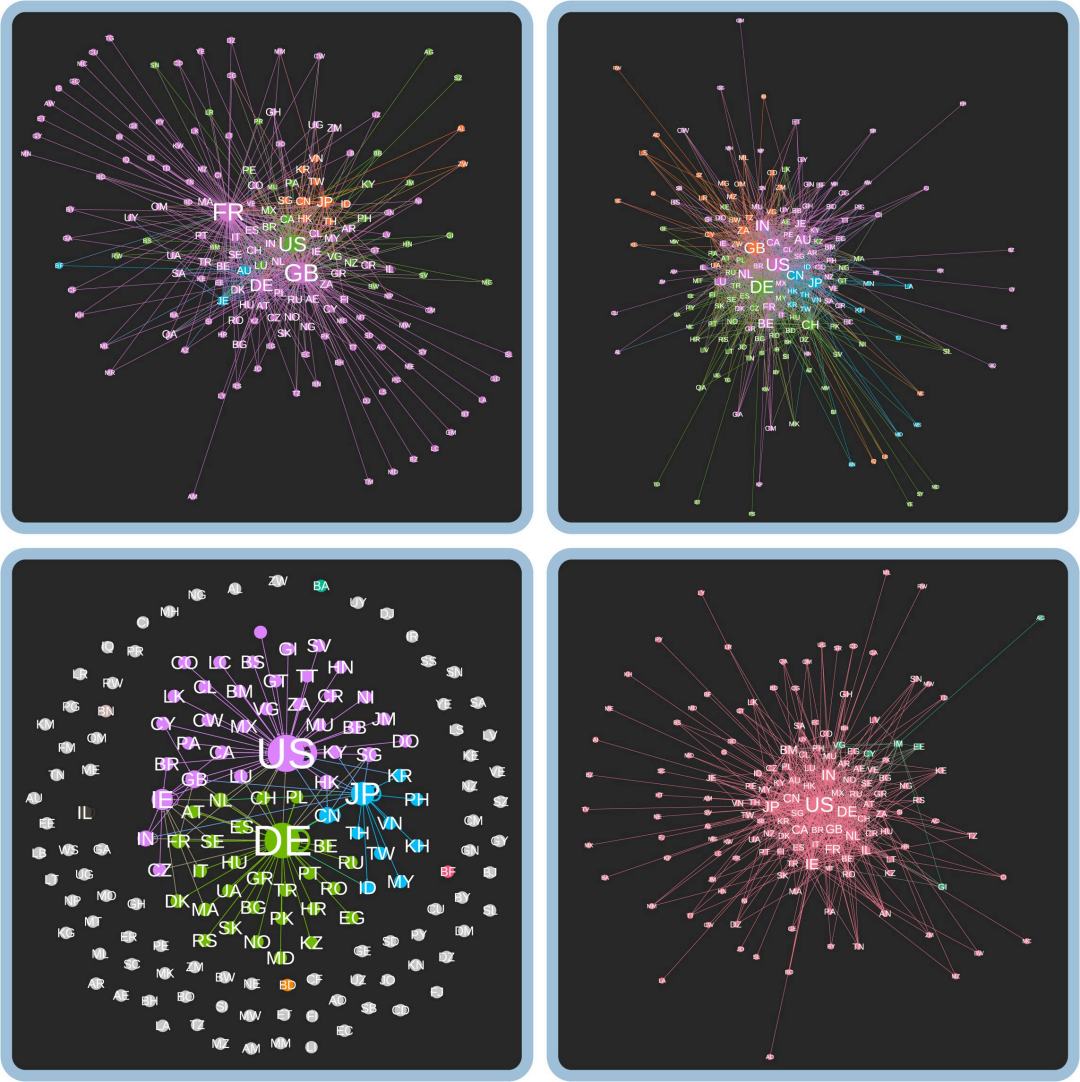
Networked industrial geographical structures of the global economy. Top-right: Technology Hardware & Equipment (clique-like), Top-left: Commercial & Professional Services (polycephalous), Bottom-left: Food & Staples Retailing (segmented-decentralised), Bottom-right: Real Estate (star-like). Colors indicate communities as partitioned by the Blondel algorithm [58], while the size of the nodes is proportional to their degree.

The resulting sector network graphs are shown in Fig 5. Each of the four conforms to one of the idealised models, including Technology Hardware & Equipment (clique-like), Commercial & Professional Services (polycephalous), Food & Staples Retailing (star-like), and Real Estate (segmented-decentralised) sectors, as explained in greater detail below. The remaining sectors are included in the supplementary materials found in the appendix.

#### Clique-like type: Technology hardware & equipment

The idealised clique-like type is exemplified by the Technology Hardware & Equipment industry network (⟨*C*⟩ = 0.70, *r* = -0.58, *D =* 0.07) (Table 2). Like in other cliques, countries in the Technology Hardware & Equipment network are tightly connected and integrated, with an average degree ⟨*k*⟩ = 9.61 (Table 2) suggesting that each country is connected to an average of 10 other countries. It includes 928 firms with 21,797 subsidiaries in 137 countries (Table 1) specialising in communications equipment, electronic equipment and components, and technology distribution and manufacturing. At the network centre of a clique is a poorly defined and less-dense ‘core’, consisting of large technology hardware producers such as the United States, China, India, Japan, Germany, France, Israel, and South Africa (Fig 5, top-right). The Technology Hardware & Equipment network features one large global community, and a smaller community consisting mainly of East Asian technology leaders (China, Japan), subsidiary producer nations (Taiwan, Thailand, Malaysia, South Korea), and a few recognised tax havens (the Cayman Islands, the British Virgin Islands). China, Japan, and the United States are home to the largest number of firms in the overall network, with firms like Tsinghua Tongfang and ZTE (China), Hitachi and Canon (Japan), Hewlett Packard and Cisco Systems (United States) each with hundreds of subsidiary locations. The role of smaller countries like Finland and Switzerland is built into the network through the subsidiaries of firms like Nokia and Logitech, respectively. The Technology Hardware & Equipment industry is thus extensively connected globally, with countries primarily embedded within a clique-like network.

#### Polycephalous type: Commercial & Professional Services sector

The sector that exemplifies the polycephalous type is Commercial & Professional Services (⟨*C*⟩ = 0.75, *r* = -0.67, ⟨*k*⟩ = 6.59, *D* = 0.04) (Table 2). It is comprised of 555 firms with 18,862 subsidiaries in 167 countries (Table 1) providing services in research, consulting, human resources, office supplies, and security. The knowledge- and service-oriented nature of this sector implies that industrial relations are reliant on face-to-face contacts [94]. This explains the relative segmentation of the network, which is organised into regional and linguistic communities (Fig 5, top-left). For instance, French firms Bureau Veritas (521 subsidiaries) and Edenred (140 subsidiaries) provide research and consulting services primarily in Africa, the Caribbean and the Pacific, linking France to its former colonies and overseas territories. The United States is the most central node in a community connecting the Americas, and also links to a global range of tax havens (e.g. the U.S. Virgin Islands and the Cayman Islands) and outsourcing hubs (e.g. the Philippines).

#### Star-like type: Food & Staples Retailing sector

Food & Staples Retailing is an example of the star-like network type (⟨*C*⟩ = 0.20, *r* = -0.73, *D* = 0.04) (Table 2). The network has an average degree of ⟨*k*⟩ = 2.96, it is one of the least ‘networked’ structures, meaning that countries in this sector are connected to just three others. It contains 175 firms with 7,279 subsidiaries in 73 countries (Table 1), specialising in retail and distribution of food and drug. There is a strong geographical component to the network structure (Fig 5, bottom-left), with the core role of the United States related to a range of food and drug retailers in the Americas, while Germany and Japan connect communities in Europe and Asia, respectively. The network’s core is dominated by large MNCs with a global presence, including Metro AG (Germany, 1393 subsidiaries), the Kroger Company (the United States, 452), Tesco (the United Kingdom, 412), Sysco Corporation (the United States, 293), and Fyffes (Ireland, 230)—all of which are involved in food wholesaling and retail. The dominance of global firms explains its core-periphery network structure in which countries on the periphery are less interconnected, generally by only one or two ties, indicating that food and staples retailing in many countries is self-sufficient with independent retailers and domestic value chains. These findings align strongly with observations of the agri-food industry outlined by Dicken [95]. In South Africa, for instance, multinational corporations tend to dominate only some subsectors, including grain storage and feedlots, with others being under control of a large number of smaller domestic economic actors [96]. The local character of produce, the perishable nature of food, and strict agricultural regulations explain the relative lack of connectivity at the network periphery.

#### Segmented-decentralised type: Real Estate

Real Estate exemplifies the segmented-decentralised network type (⟨*C*⟩ = 0.6447, *r* = -0.571, *D* = 0.06) (Table 2). The industry network connects 105 countries via ties of 868 firms with 51,876 subsidiaries (Table 1) operating across two major subsectors, including real estate investment trusts (REITs) and real estate management & development. Countries in this sector are connected with an average degree ⟨*k*⟩ = 5.70. It is the least hierarchically differentiated network among 24 industry sectors, and is characterised by geographically segmented subnetworks, with a large global community, a European community, and another spanning Asia and East/Central Europe. This explained by the fact that Real Estate is tied to intensely localised property markets, and thus firm activity reflects the financialisation of the industry more than production or consumption itself, with tax havens and offshore financial centres being prominent in the network (Jersey, the British Virgin Islands, the Cayman Islands, Cyprus, and Bermuda).

## Discussion and conclusion

As the global economy becomes more integrated through multinational firm activity, analysis of sectoral variation in global networks reveals the ‘structural architecture’ that underlies international economic structures. The characterisation of the different sectors, and the position of national economies, within global networks can be better achieved through a refined understanding of the processes and practices that scaffold the global economy [4, 52, 56].

This paper has developed a novel method for comparing international economic networks by sector based on the ownership linkages of MNCs. How MNCs have connected across space has been long associated in studies of globalisation and internationalisation with differences in how specific sectors operate [95] or how individual firms internationalise to coordinate various trade-related activities [97]. The characterisation of sectoral networks into four idealised network types (‘Segmented-Decentralised’, ‘Polycephalous’, ‘Star-like’, and ‘Clique-like’) advances beyond individual attempts to clarify industry-specific globalisations, including Braham and Mensi [97] and Saarenketo et al. [98] exploring Telecommunication Services; Bonaglia et al. [99] exploring Capital Goods; and Ekeledo and Sivakumar [100] for Manufacturing and Service firms.

Based on the analysis, we add to the literature on variation by industry sector in shaping multiple globalisations [13, 16] in city networks by focussing on variation across sectors using international networks. The sectoral variation we identify in international economic networks combines network metrics in order to move beyond analyses applying centrality measures and community detection.

Sectors with a relatively high average clustering coefficient ⟨*C*⟩ often form dense, interconnected networks with regionalised components, which we argue can be classified into either polycephalous or clique-like types. The international networks of Capital Goods, Commercial & Professional Services, Materials, Pharmaceuticals, Biotechnology & Life Sciences are all polycephalous, while other highly clustered international networks are Food, Beverage & Tobacco and Technology Hardware & Equipment, whose networks are clique-like. Both polycephalous and clique-like types reflect tightly bound global networks, with the difference being that the former are more hierarchically differentiated in nature, featuring greater disassortativity within international firm networks. This aligns with the findings in the ‘white goods’ sector [99], with a very strongly connected network of market leaders is linked to firms in more peripheral locations involved in component production or in distribution and sales in high growth emerging markets. Similarly, the clustered organisation of firms in countries involved in extractive sectors (materials) [95, 101] is explained by peripheral but strongly networked resource production and extraction locations connecting to consumer markets across the global economy. Highly global, but less hierarchically differentiated, clique-like structures were also documented agro-food industry [95]—a highly local economic activity with increasingly global distribution through global production chains.

Sectors with a relatively low average clustering coefficient ⟨*C*⟩ often operate within more regional, or local, subnetworks. Segmented-decentralised international industry structures such as Real Estate, Energy, and Telecommunication Services—although global in scope—have fundamentally regionalised components, for example, based on the relationships between suppliers, manufacturers, and distributors. The role of large state-owned, and state-related, enterprises in certain energy [101] and telecommunications [102] sub-sectors is particularly apparent, explained by a relatively high degree of regulation that precludes extensive globalisation. Star-like sector networks, such as Food & Staples Retailing, Automobiles & Components, and Retailing, feature relatively higher centralisation as they have a more well-defined network core despite low overall network density due to a high number of less globally extensive connected countries. The difference between segmented-decentralised and star-like networks is the degree to which some sectors, such as Banks (star-like structures), are hierarchically differentiated, whereas Real Estate, Energy, Consumer Services, and Utilities (segmented-decentralised) networks include many weakly connected countries. Indeed, as outlined by Wrigley [103], the star-like structure of Food & Staples Retailing and Retailing sectors may be the result of global consolidation since the 1990s by leading firms through the mergers and acquisitions of smaller firms.

The results suggest that both firm- and country-level dynamics explain the degree to which a sector is more or less extensively globally connected. A multilayer network perspective shows that countries may be specialised in some sectors but not in others, while a small number of countries comprise the core of most networks. Such findings provide additional insights into observations that economic specialisation is responsible in part for globalisation at the national scale, as are economies of scale that produce externalities tied to the size of a national economy. Thus, larger economies such as the United States, Germany, Japan, and China are often highly central to networks while smaller economies are often less central and less extensively connected across the world. Exogenous orientation explains much of the positionality within the international network, particularly in countries whose position is core to some sectors but not others. As Breul [104], Martinus et al. [64] and others have argued, small states in particular pursue internationalisation strategies around key sectors. For example, some countries containing some well-known tax havens and offshore financial centres are placed in the network core only if the interplay between the different sectors is considered (multiplex core-periphery). That niche economic functions such as tax havens also shape the network structures [77] confirms that economic activity alone is insufficient to explain networks resulting from global ownership structures, and that financialisation must increasingly be considered as a primary driver of cross-border relationships forged by MNCs.

Ultimately, the typology of four idealised networks provides a framework for understanding variation in the structural architecture of international networks by sector. Though alternative network-level measures may be substituted, we find that the combination of clustering and assortativity helps explain core-periphery structures by sector, particularly insofar as they can be global in multiple ways. Some sectors feature relatively well-defined core that may be rather more (e.g. Technology Hardware & Equipment, Materials) or less (e.g. Energy, Utilities) globally connected. We find that advanced service sectors (e.g. Banks, Insurance) exhibit strong hierarchical differentiation through extensive ‘global’ networks, while others are less clustered and therefore are characterised as ‘localised’ and ‘regionalised’ sectors. When combined, the typological framework helps identify sectoral variation in the structural architecture of international economic networks, which serves as a critical dimension to understanding and conceptualising how multiple globalisations are shaped by the large number of complex intraorganisational relationships.

## Supporting information

S1 Fig Network graph of Countries: Automobiles & Components. (TIF)

S2 Fig Network graph of Countries: Banks. (TIF)

S3 Fig Network graph of Countries: Capital Goods. (TIF)

S4 Fig Network graph of Countries: Commercial & Professional Services. (TIF)

S5 Fig Network graph of Countries: Consumer Durables & Apparel. (TIF)

S6 Fig Network graph of Countries: Consumer Services. (TIF)

S7 Fig Network graph of Countries: Diversified Financials. (TIF)

S8 Fig Network graph of Countries: Energy. (TIF)

S9 Fig Network graph of Countries: Food & Staples Retailing. (TIF)

S10 Fig Network graph of Countries: Food, Beverage & Tobacco. (TIF)

S11 Fig Network graph of Countries: Health Care Equipment & Services. (TIF)

S12 Fig Network graph of Countries: Household & Personal Products. (TIF)

S13 Fig Network graph of Countries: Insurance. (TIF)

S14 Fig Network graph of Countries: Materials. (TIF)

S15 Fig Network graph of Countries: Media. (TIF)

S16 Fig Network graph of Countries: Pharmaceuticals, Biotechnology & Life Sciences. (TIF)

S17 Fig Network graph of Countries: Real Estate. (TIF)

S18 Fig Network graph of Countries: Retailing. (TIF)

S19 Fig Network graph of Countries: Semiconductors & Semiconductor Equipment. (TIF)

S20 Fig Network graph of Countries: Software & Services. (TIF)

S21 Fig Network graph of Countries: Technology Hardware & Equipment. (TIF)

S22 Fig Network graph of Countries: Telecommunication Services. (TIF)

S23 Fig Network graph of Countries: Transportation. (TIF)

S24 Fig Network graph of Countries: Utilities. (TIF)
