# Supplementary figures and images for: The structural architecture of international industry networks in the global economy
Source: PLoS One. 2021 Aug 16;16(8):e0255450. doi: 10.1371/journal.pone.0255450 (PMC8366998; doi:10.1371/journal.pone.0255450)

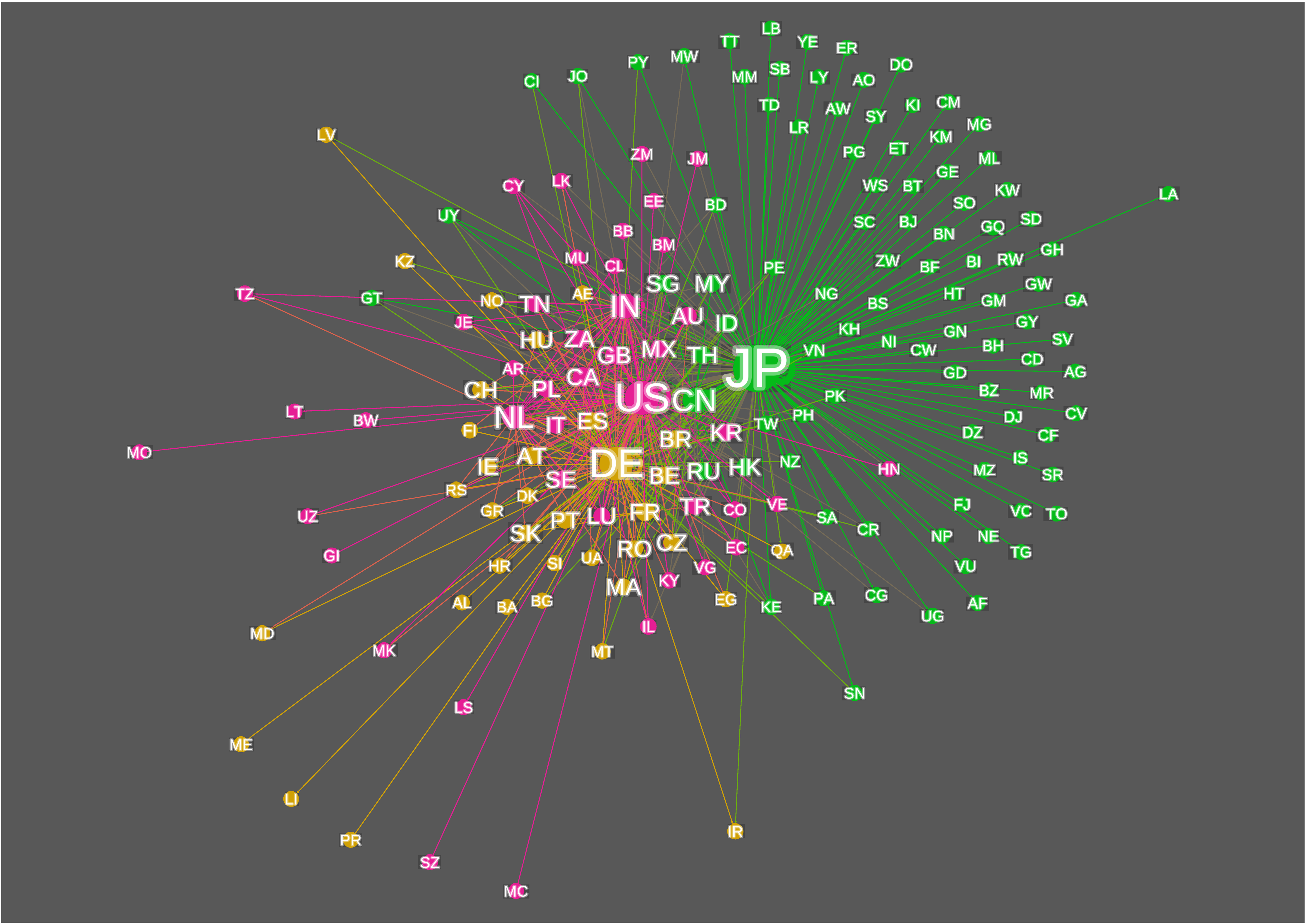

Supplement: S1 Fig — (TIF) [file pone.0255450.s001.tif]

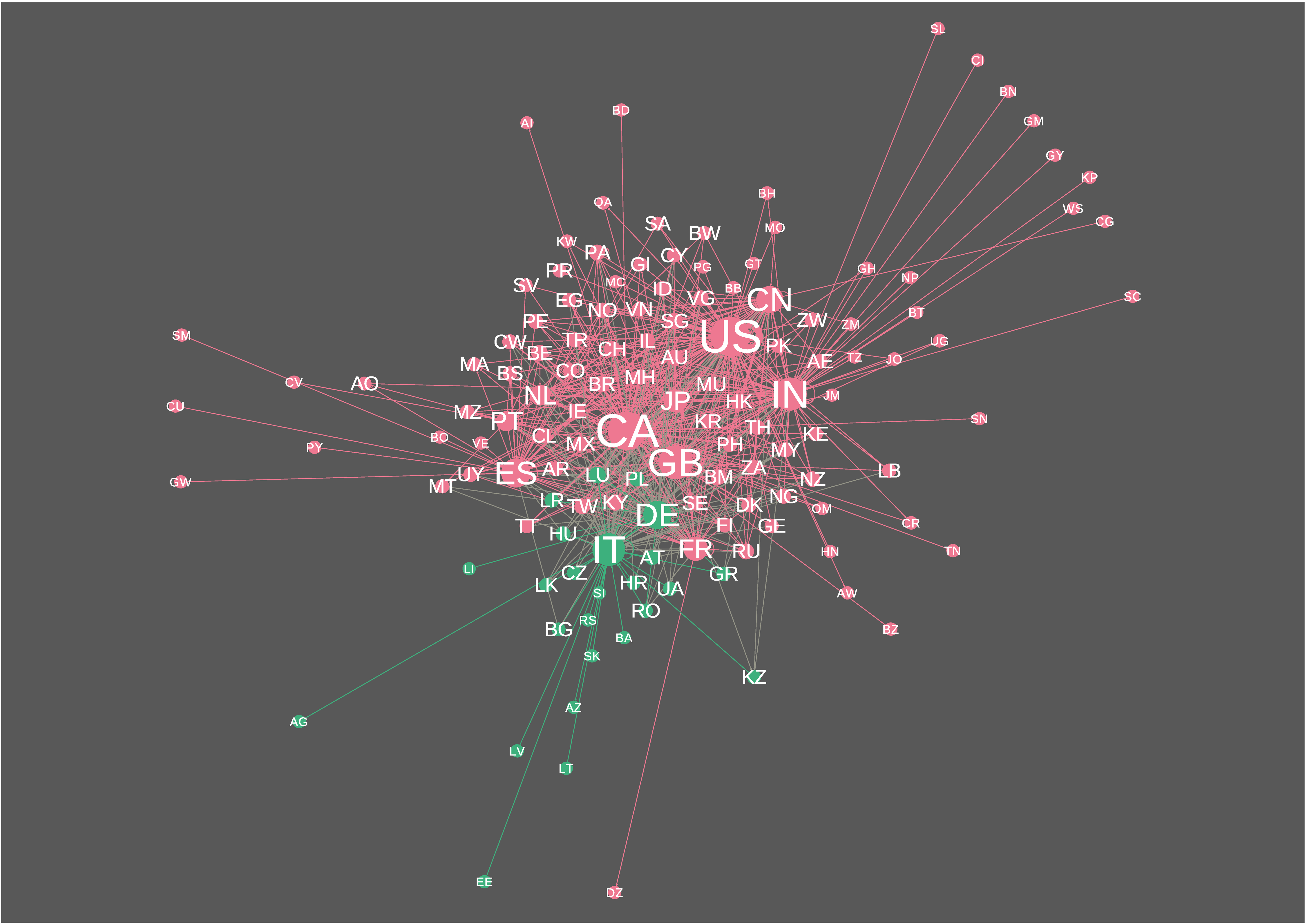

Supplement: S2 Fig — (TIF) [file pone.0255450.s002.tif]

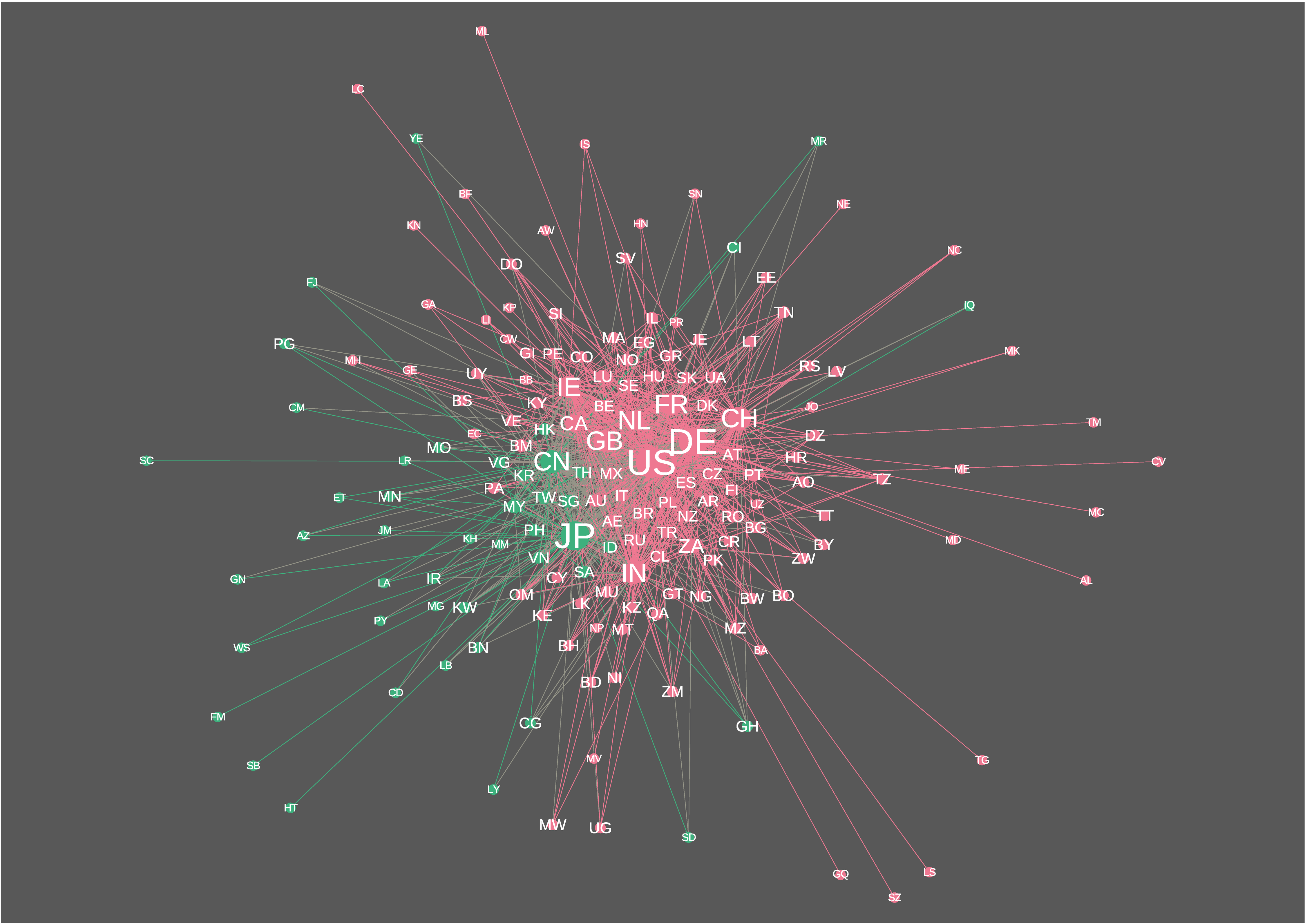

Supplement: S3 Fig — (TIF) [file pone.0255450.s003.tif]

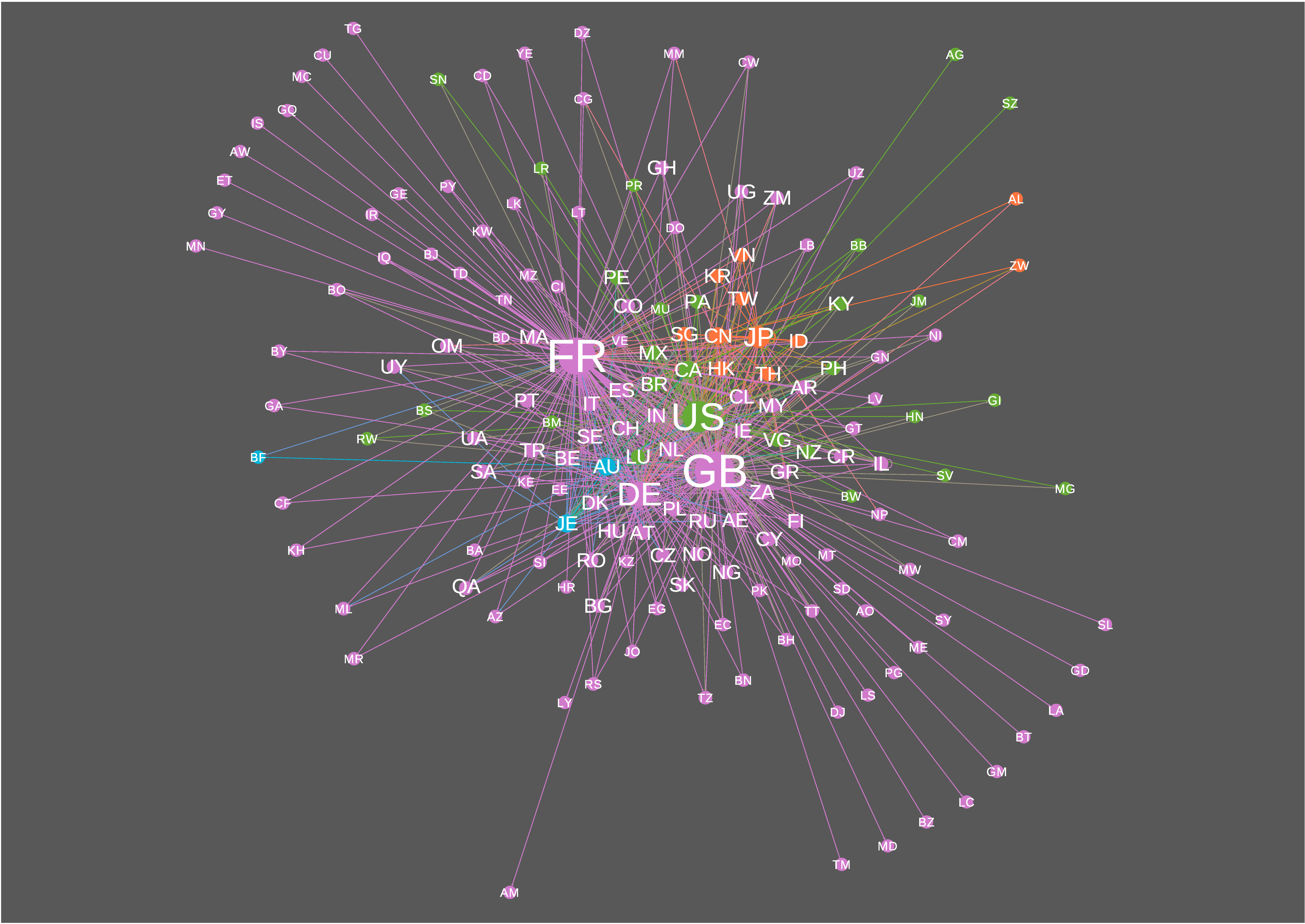

Supplement: S4 Fig — (TIF) [file pone.0255450.s004.tif]

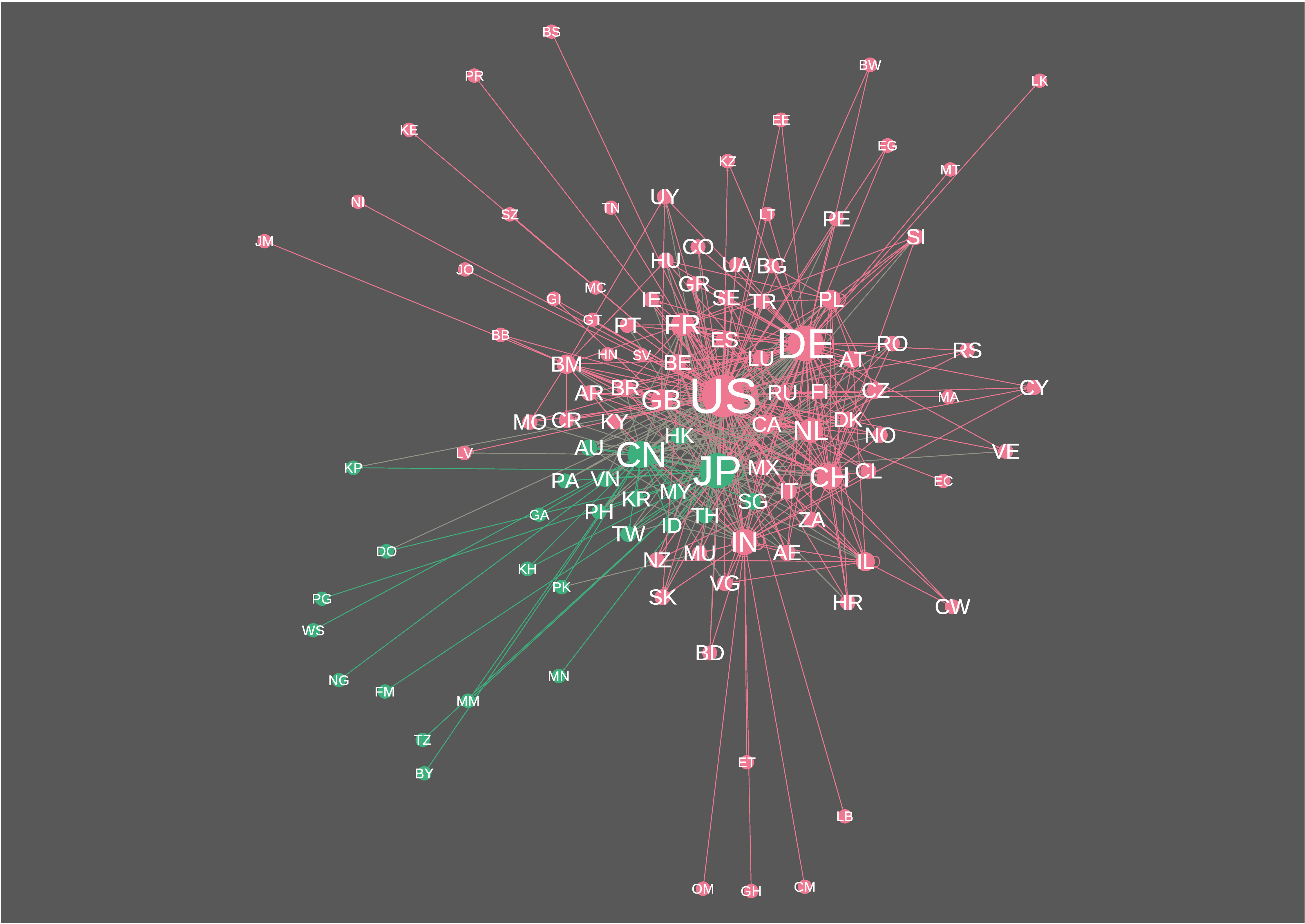

Supplement: S5 Fig — (TIF) [file pone.0255450.s005.tif]

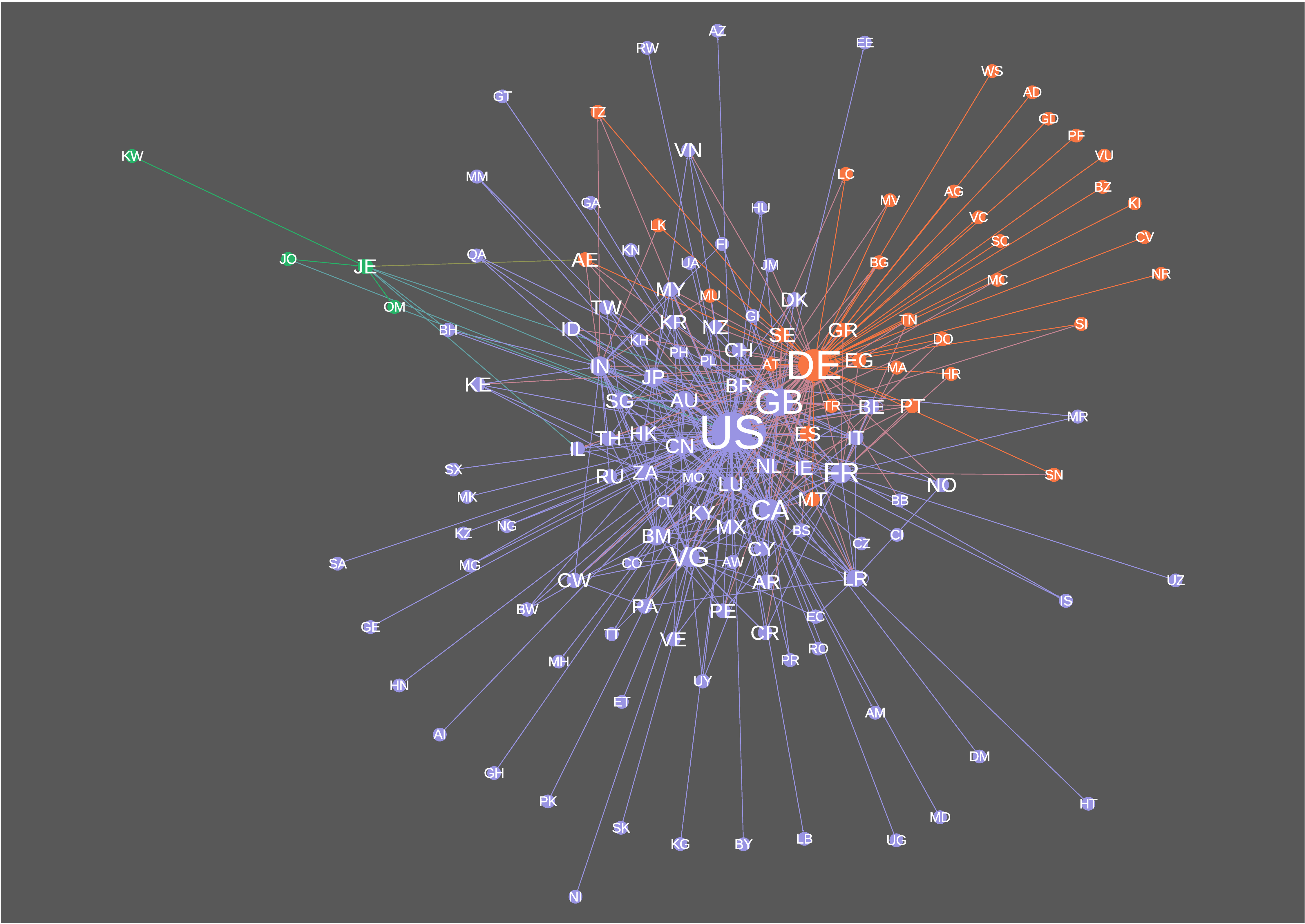

Supplement: S6 Fig — (TIF) [file pone.0255450.s006.tif]

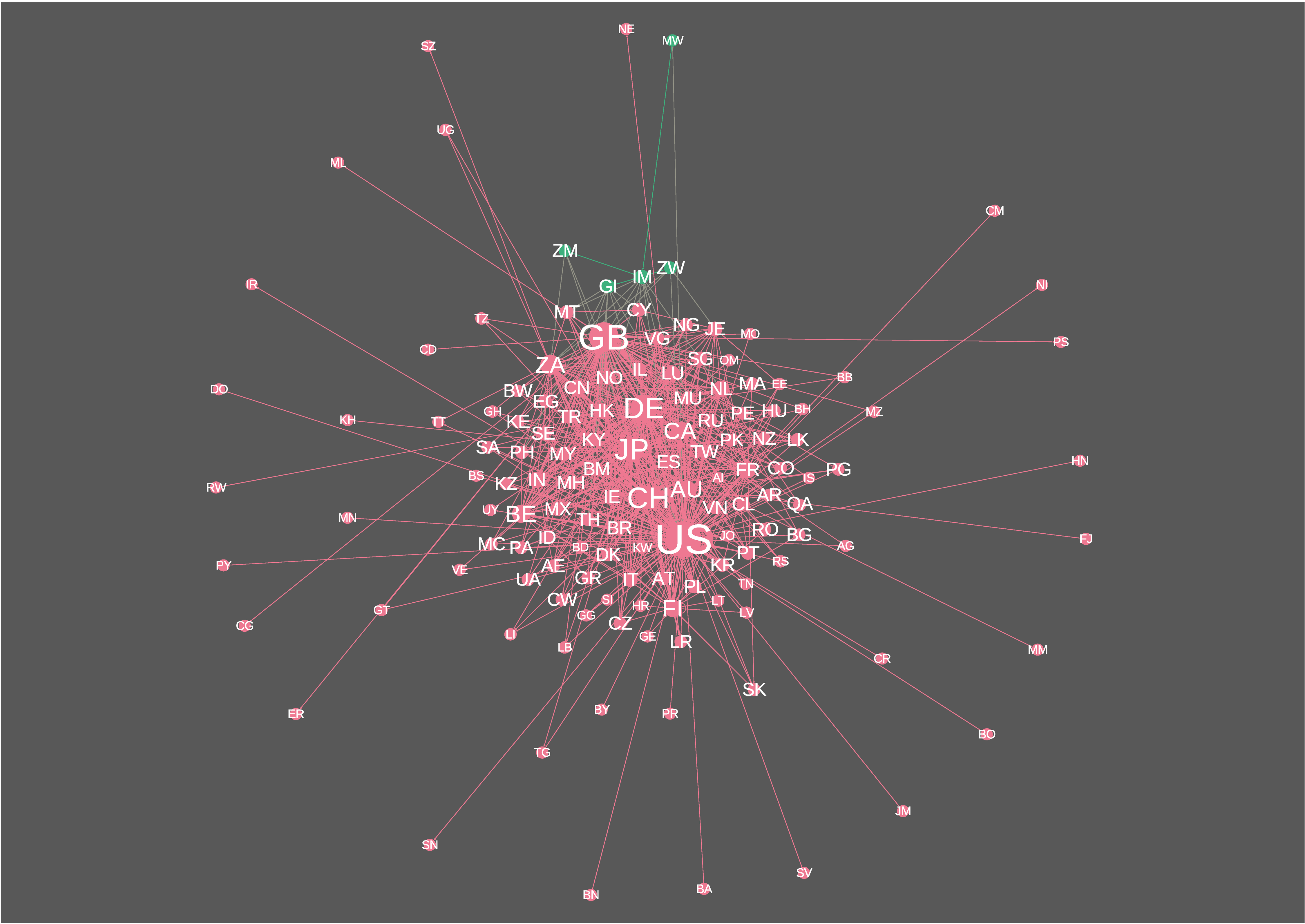

Supplement: S7 Fig — (TIF) [file pone.0255450.s007.tif]

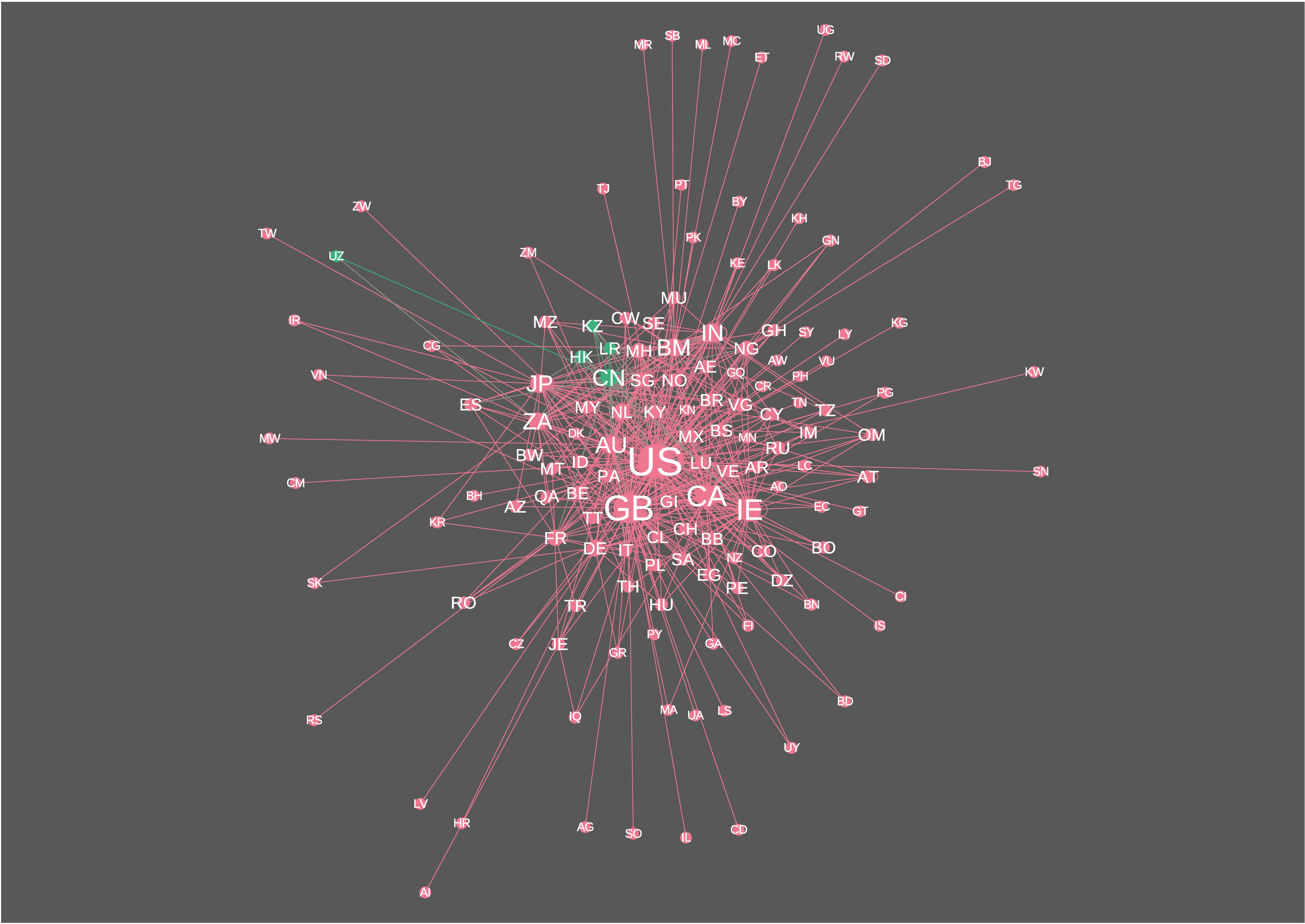

Supplement: S8 Fig — (TIF) [file pone.0255450.s008.tif]

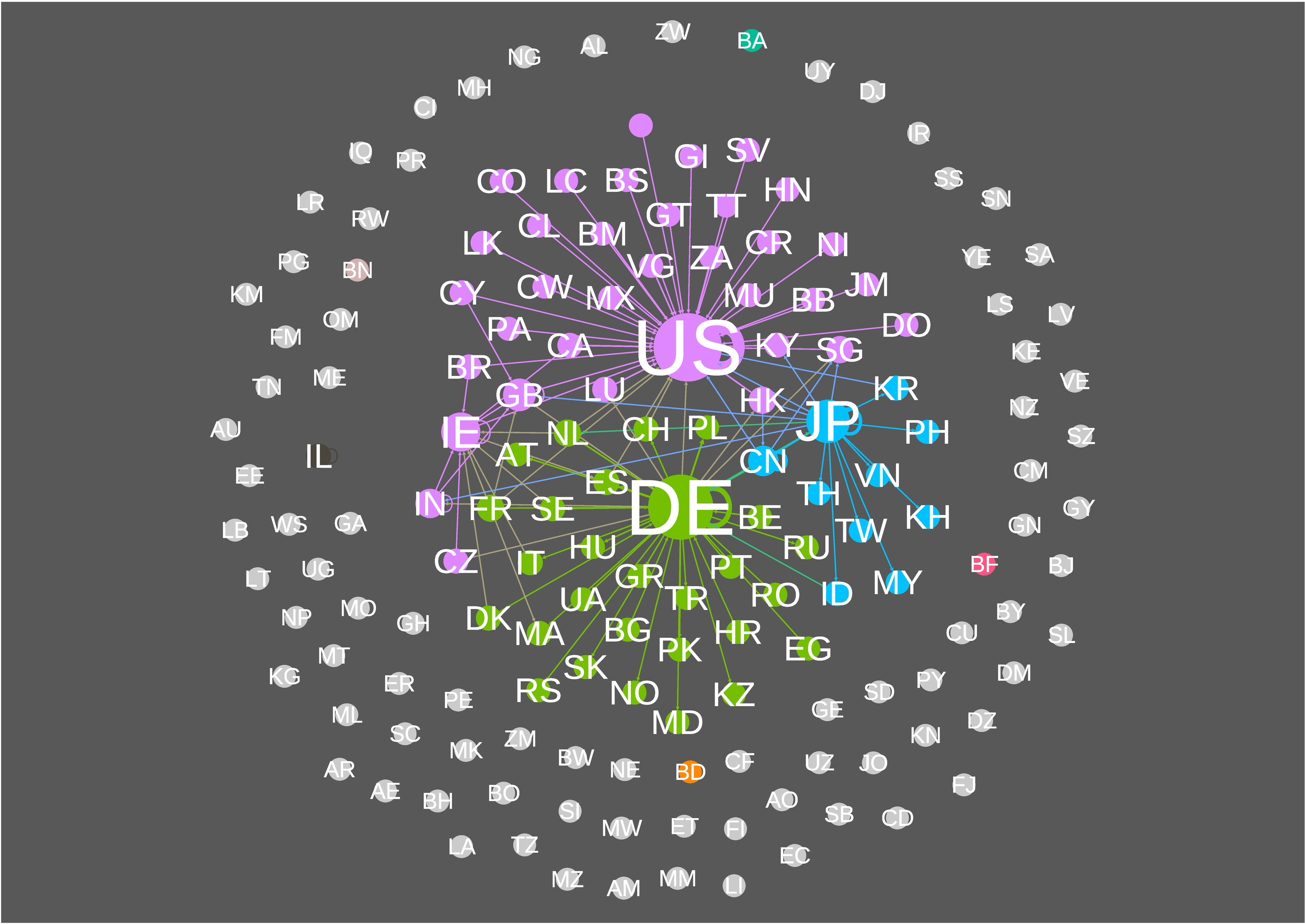

Supplement: S9 Fig — (TIF) [file pone.0255450.s009.tif]

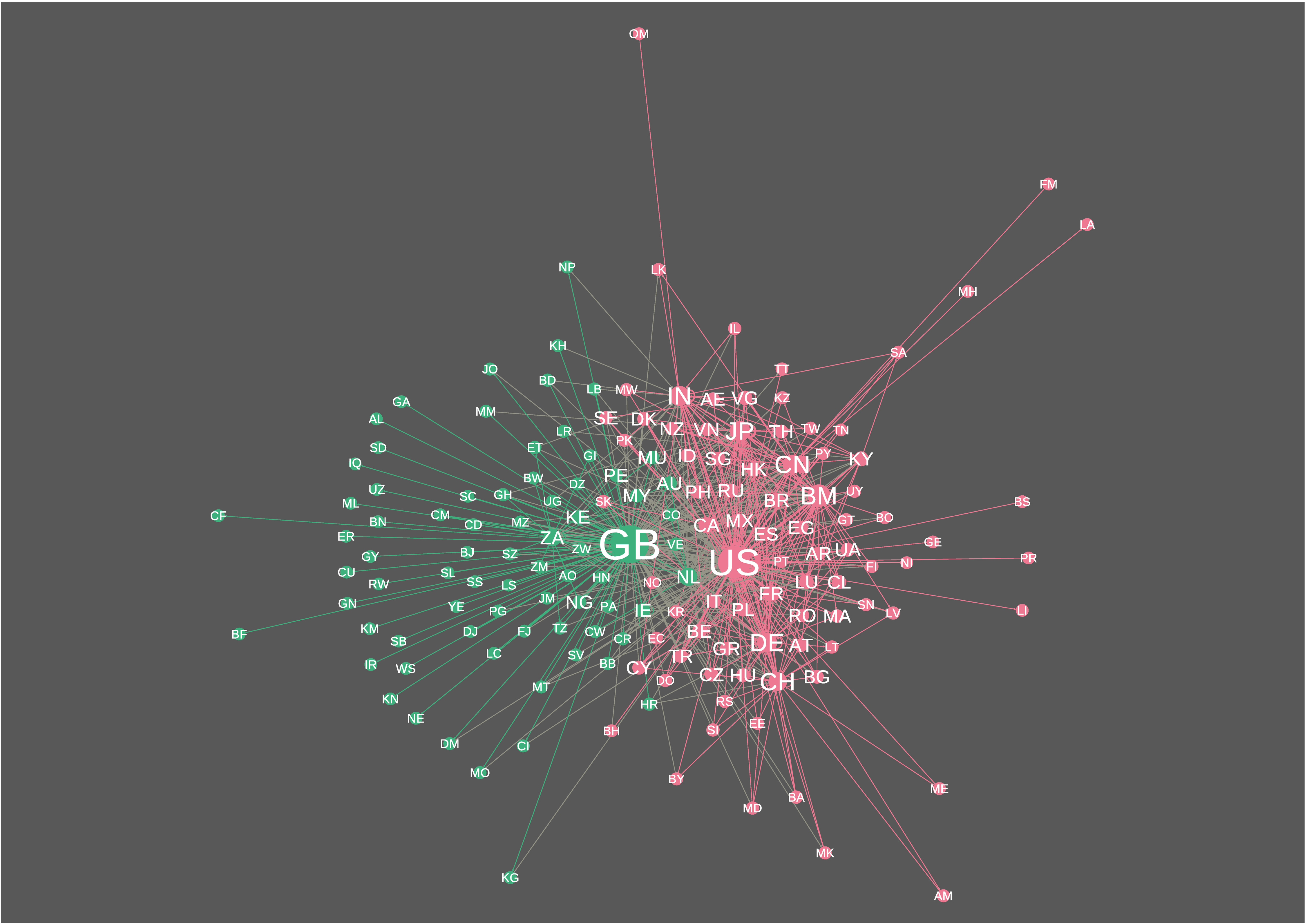

Supplement: S10 Fig — (TIF) [file pone.0255450.s010.tif]

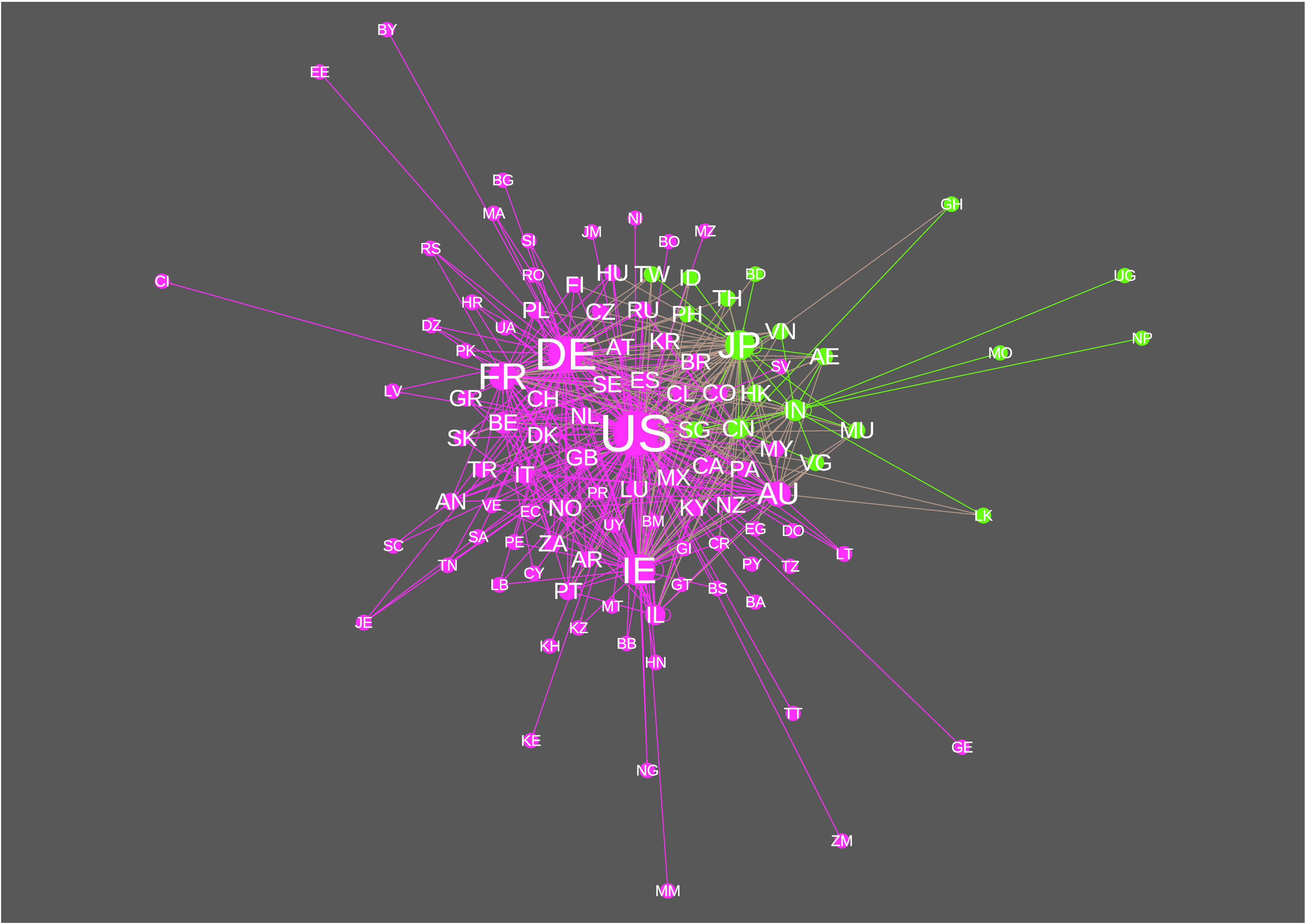

Supplement: S11 Fig — (TIF) [file pone.0255450.s011.tif]

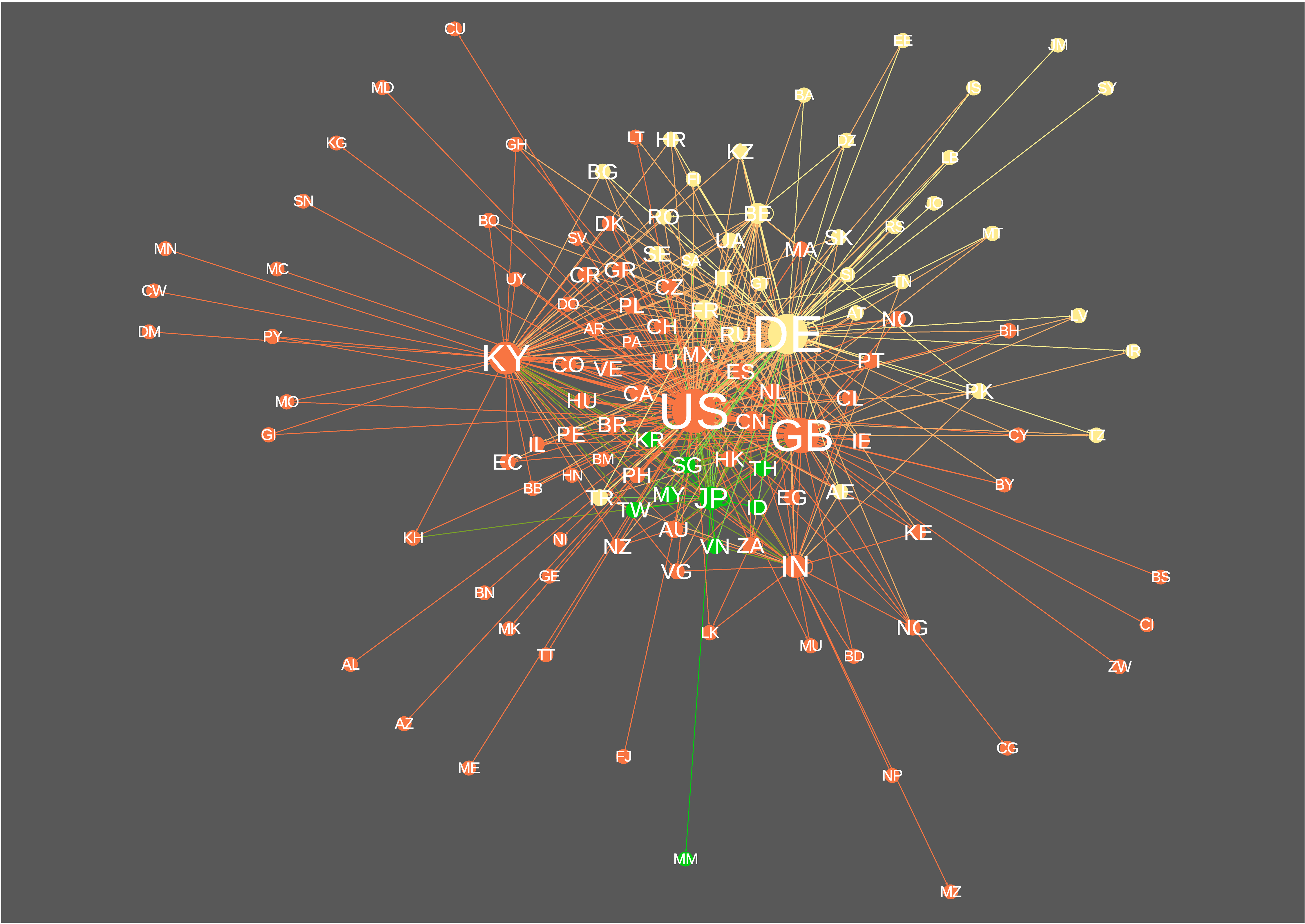

Supplement: S12 Fig — (TIF) [file pone.0255450.s012.tif]

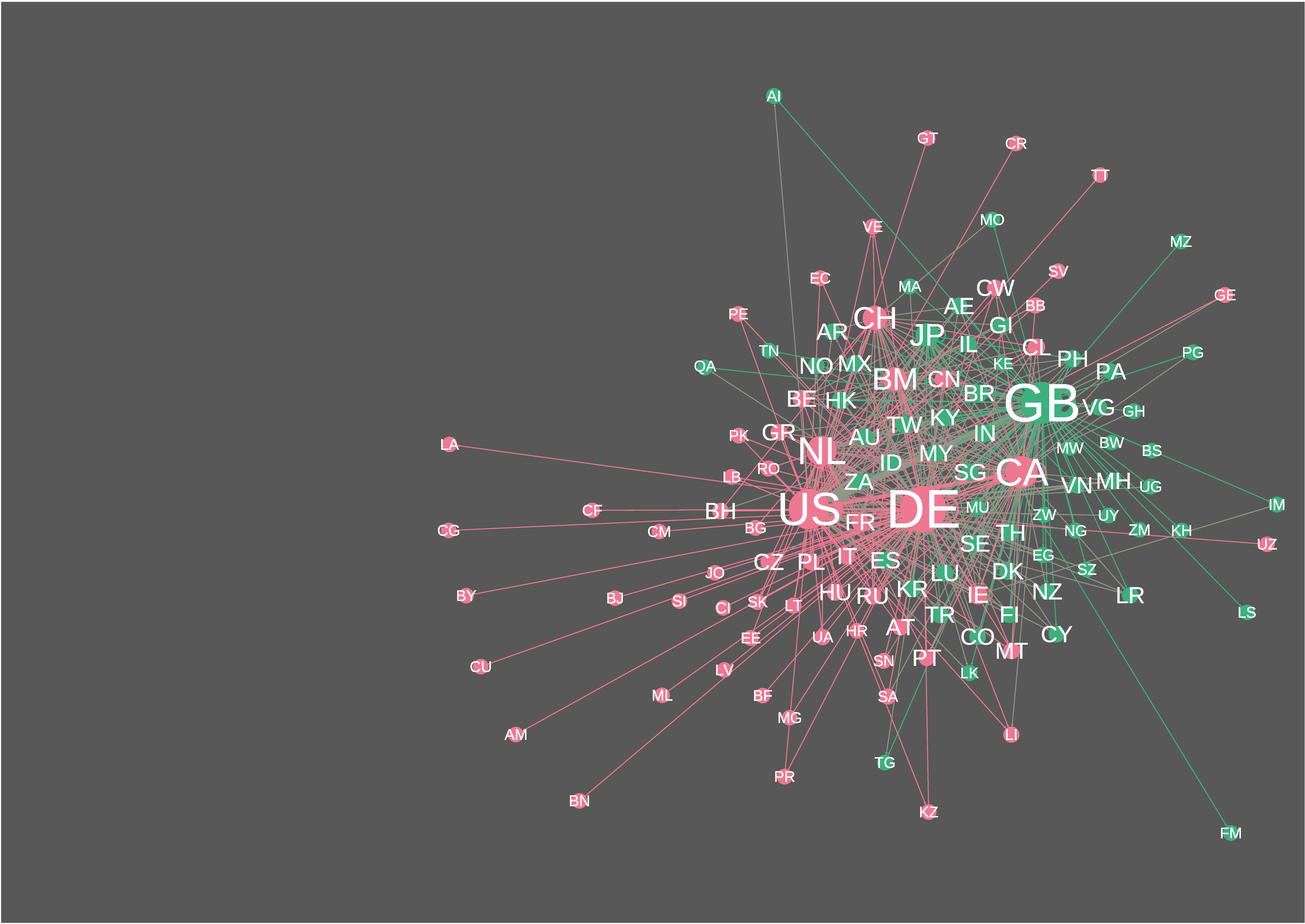

Supplement: S13 Fig — (TIF) [file pone.0255450.s013.tif]

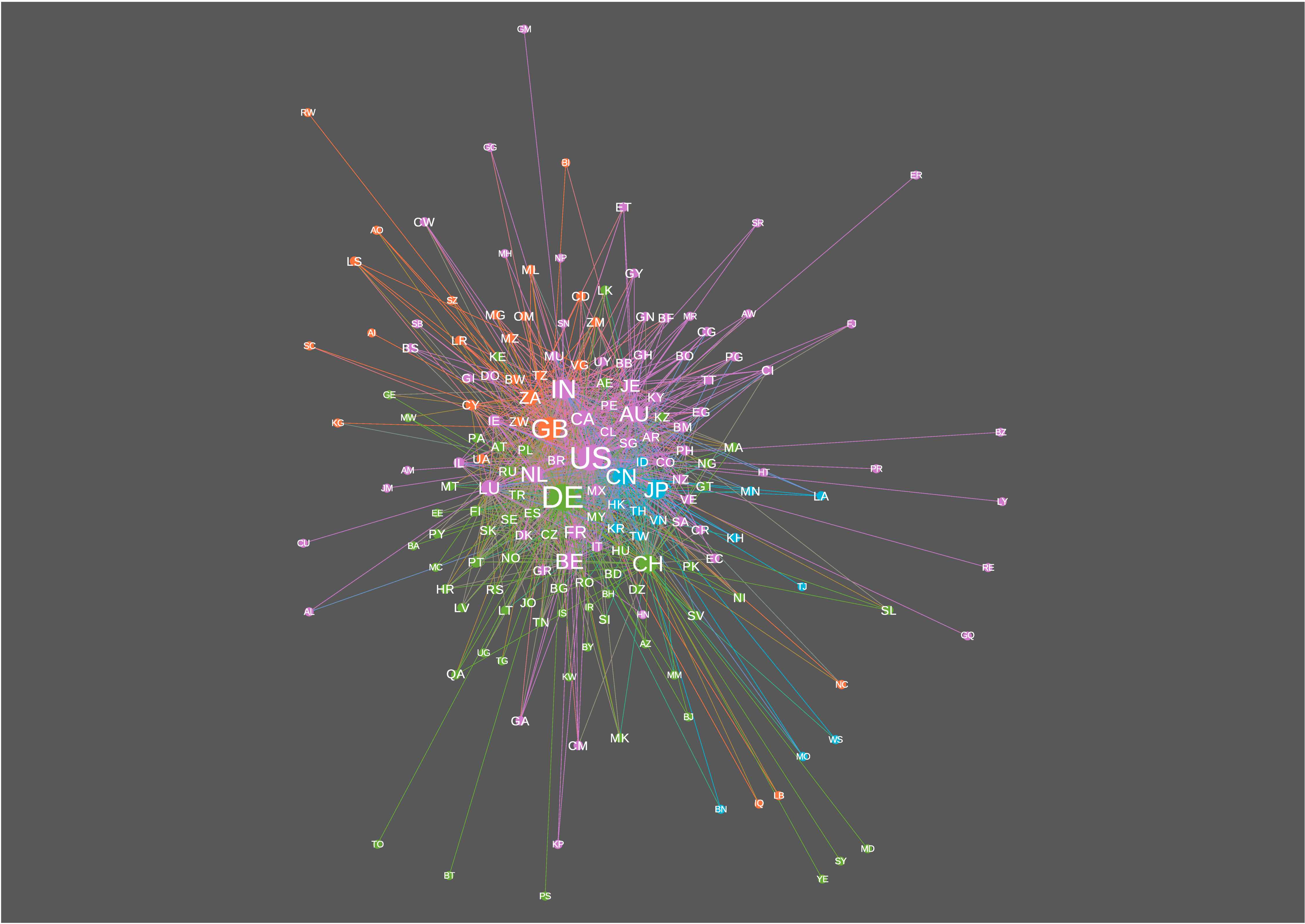

Supplement: S14 Fig — (TIF) [file pone.0255450.s014.tif]

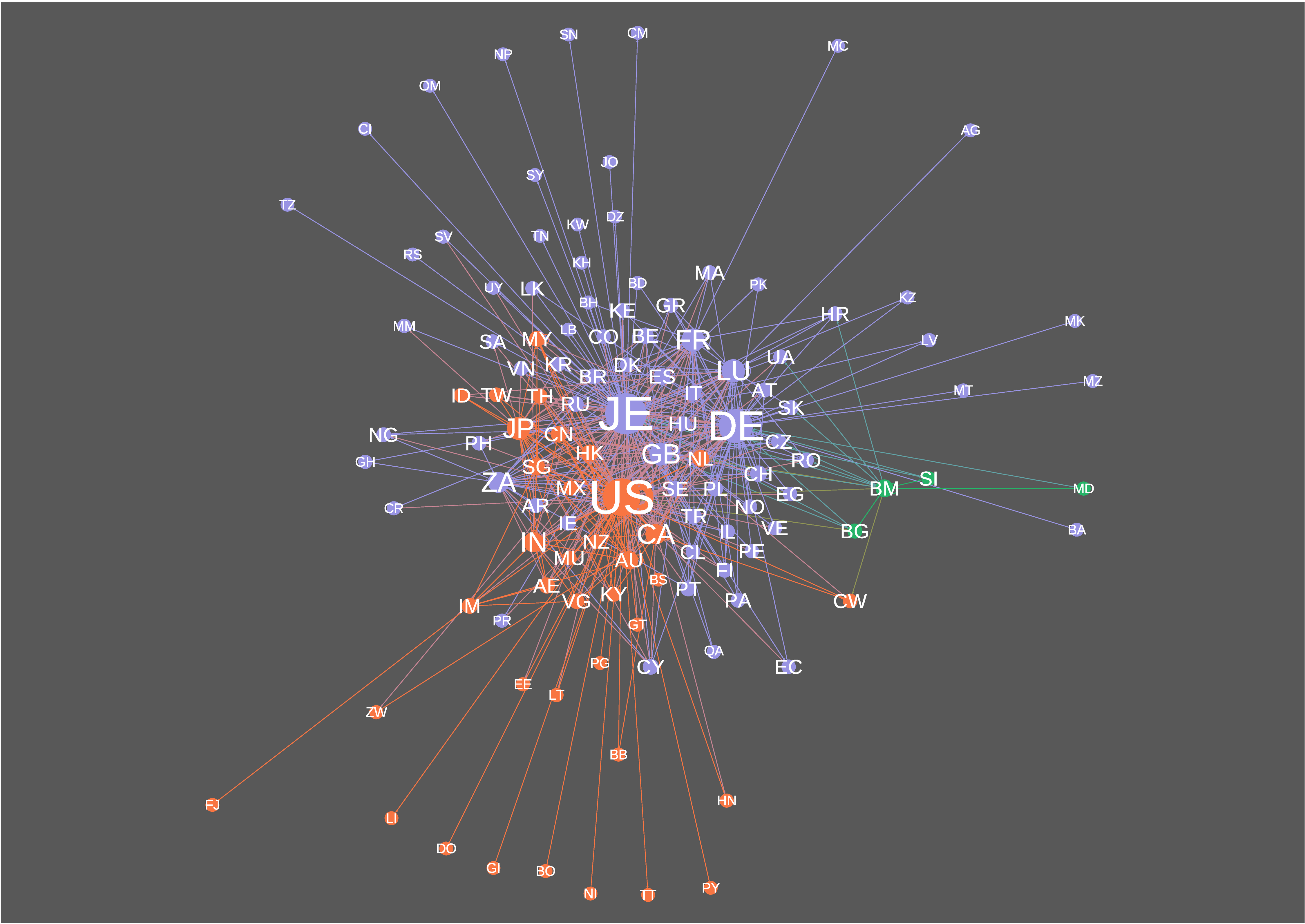

Supplement: S15 Fig — (TIF) [file pone.0255450.s015.tif]

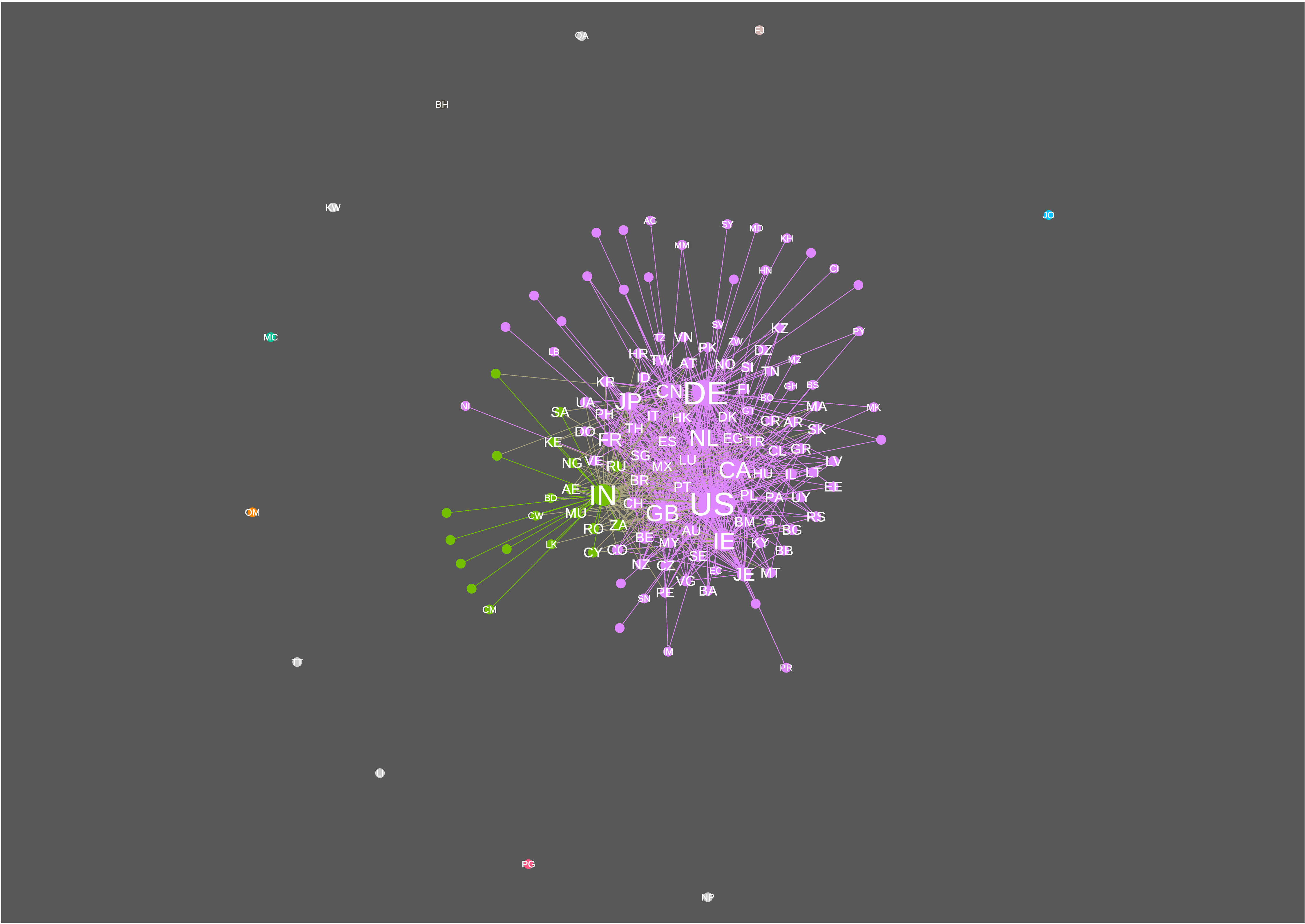

Supplement: S16 Fig — (TIF) [file pone.0255450.s016.tif]

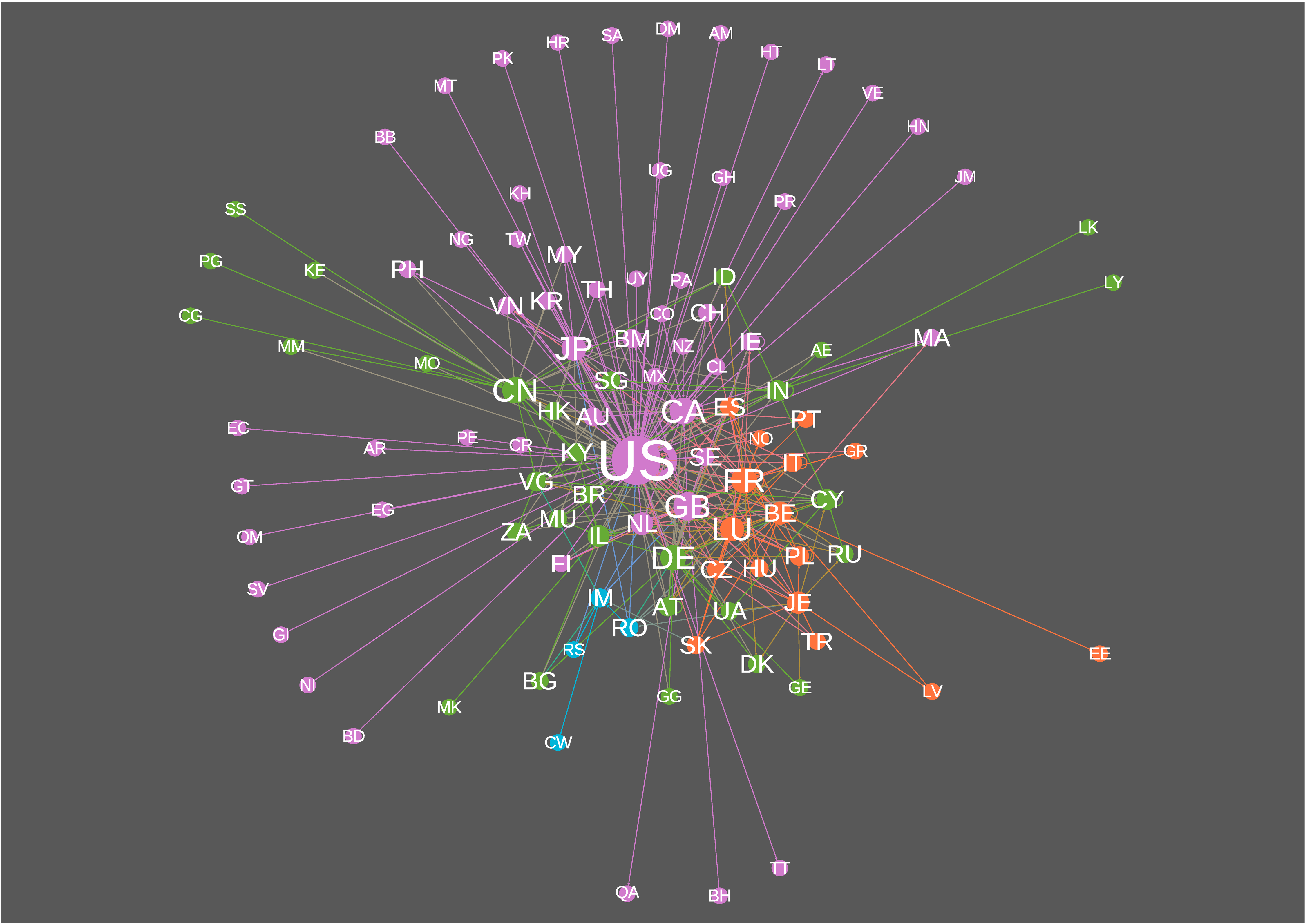

Supplement: S17 Fig — (TIF) [file pone.0255450.s017.tif]

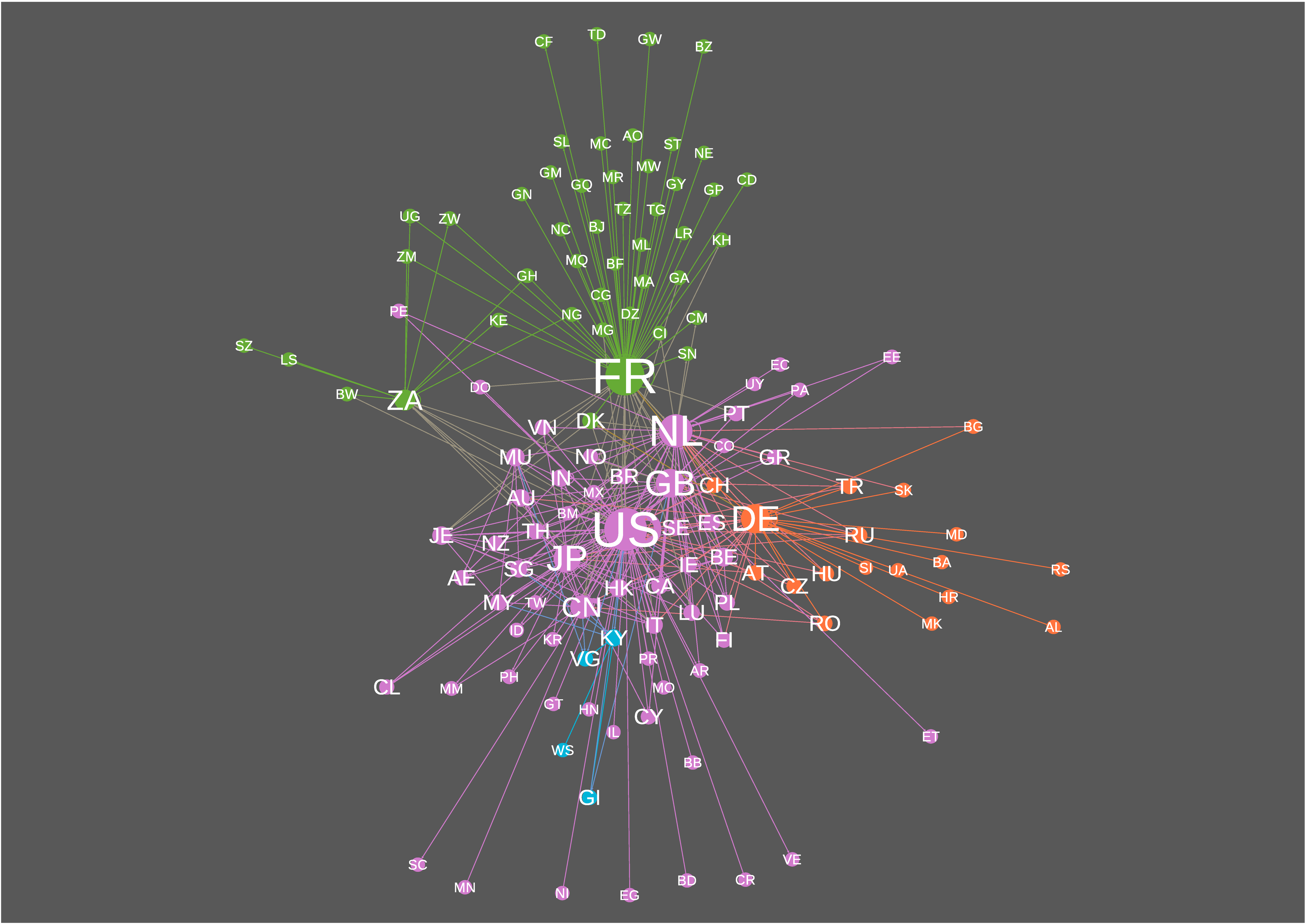

Supplement: S18 Fig — (TIF) [file pone.0255450.s018.tif]

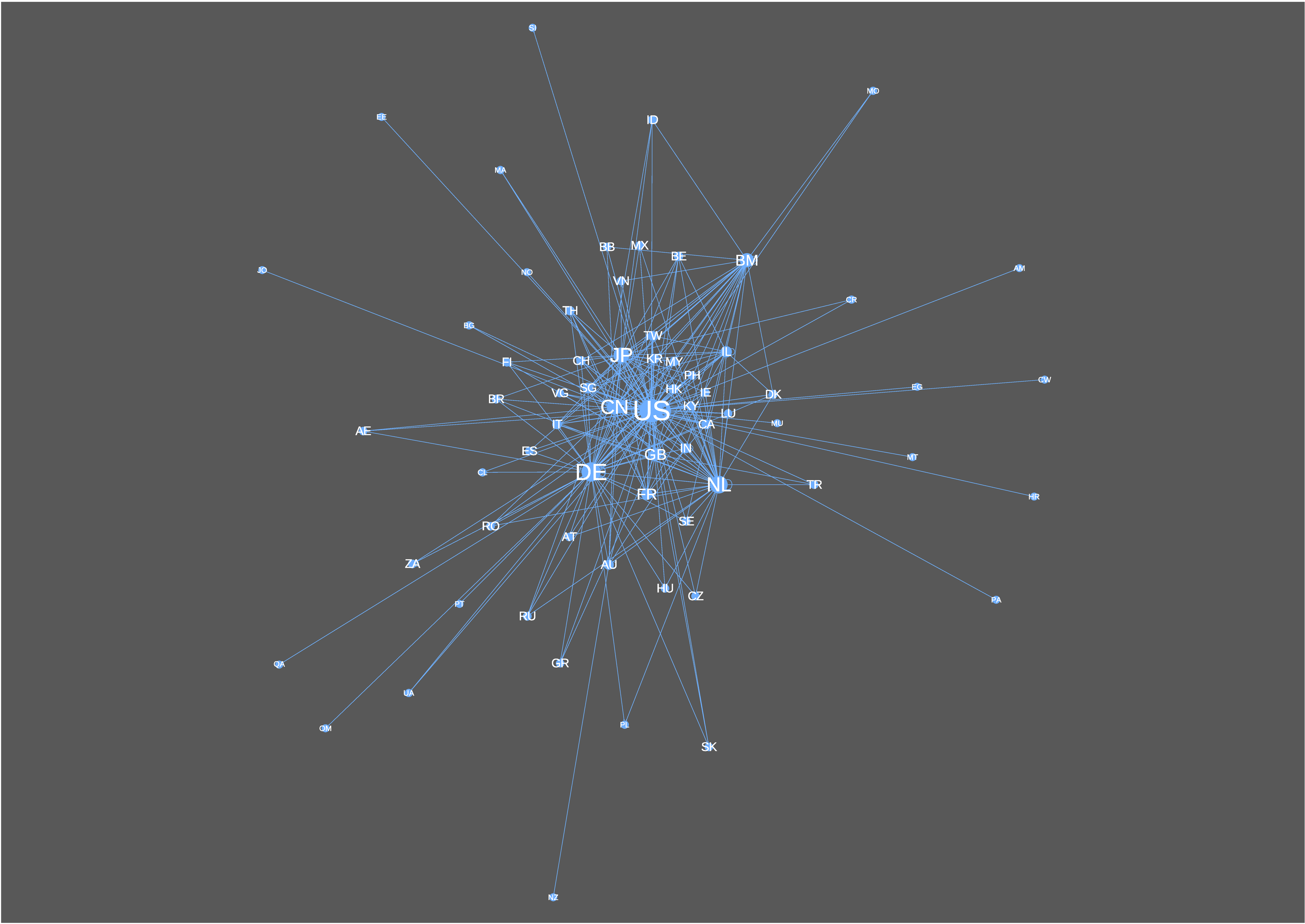

Supplement: S19 Fig — (TIF) [file pone.0255450.s019.tif]

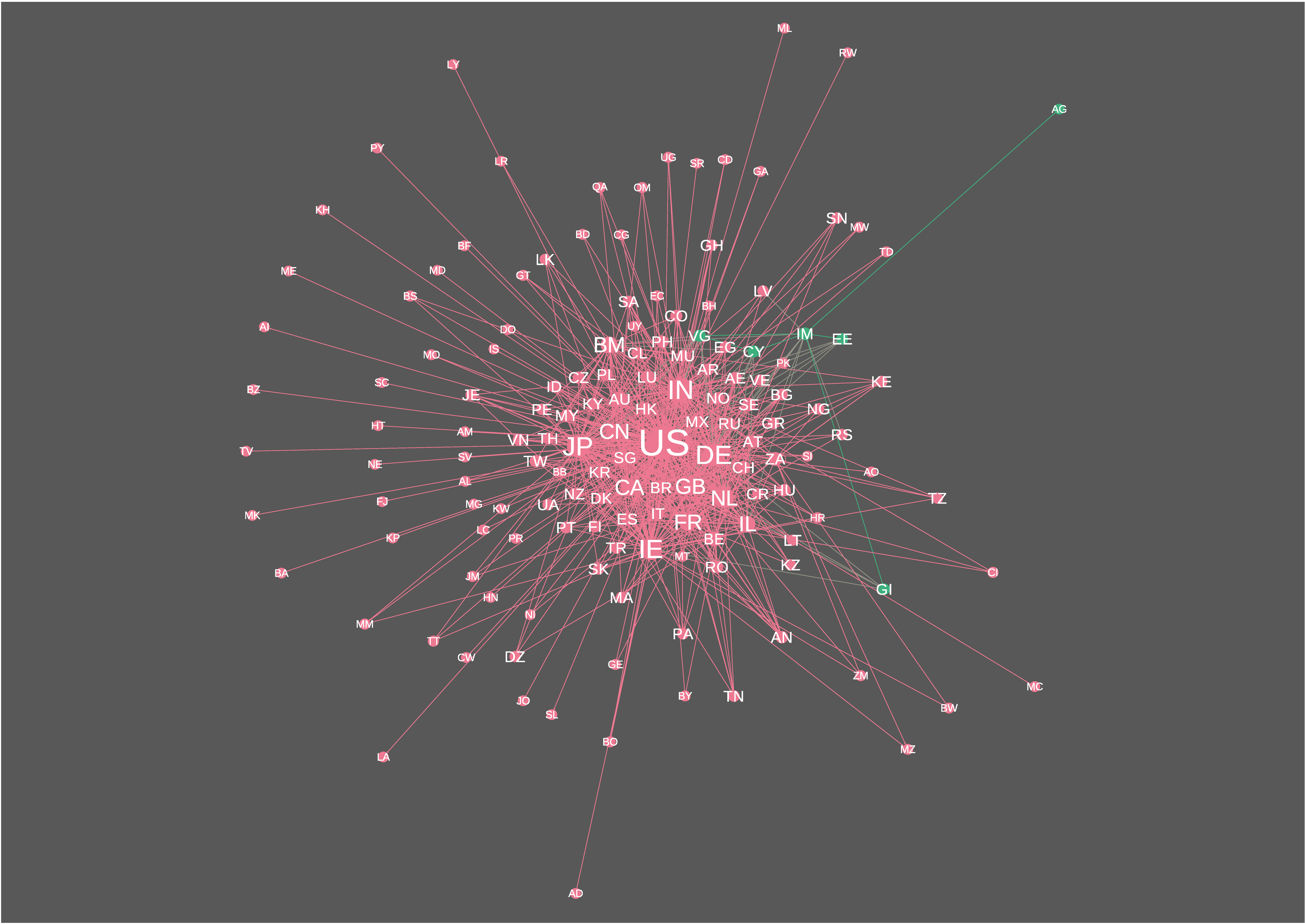

Supplement: S20 Fig — (TIF) [file pone.0255450.s020.tif]

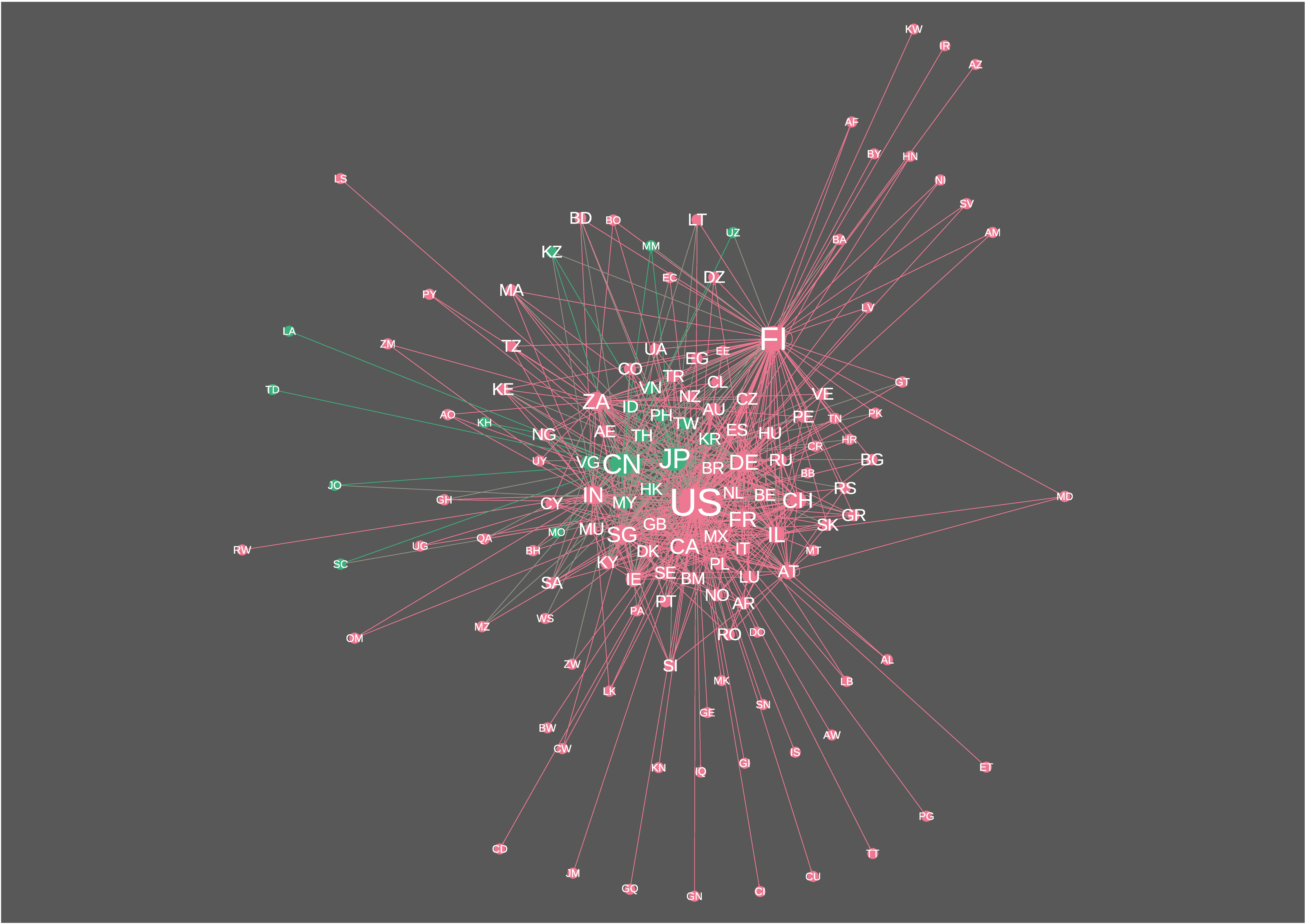

Supplement: S21 Fig — (TIF) [file pone.0255450.s021.tif]

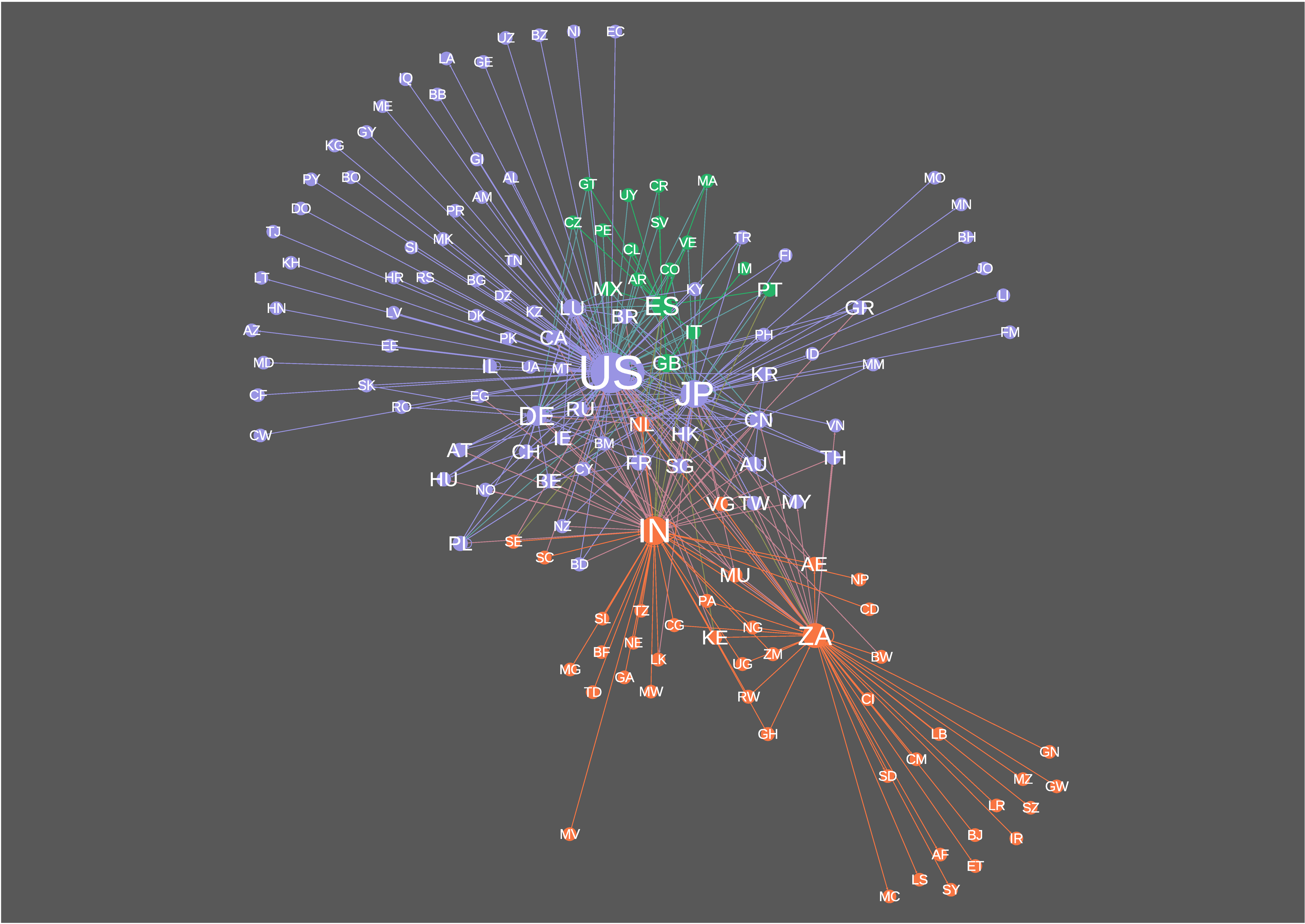

Supplement: S22 Fig — (TIF) [file pone.0255450.s022.tif]

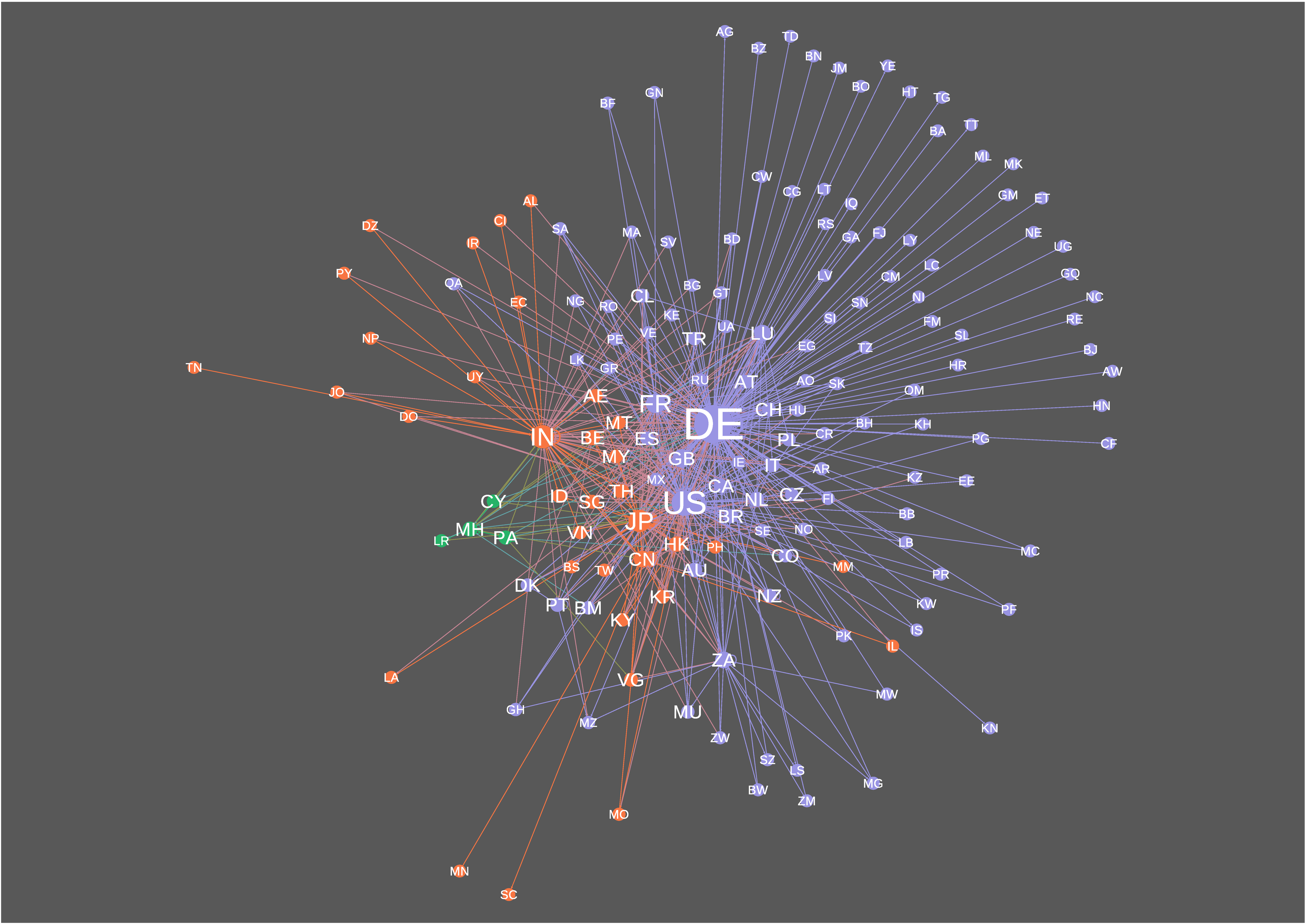

Supplement: S23 Fig — (TIF) [file pone.0255450.s023.tif]

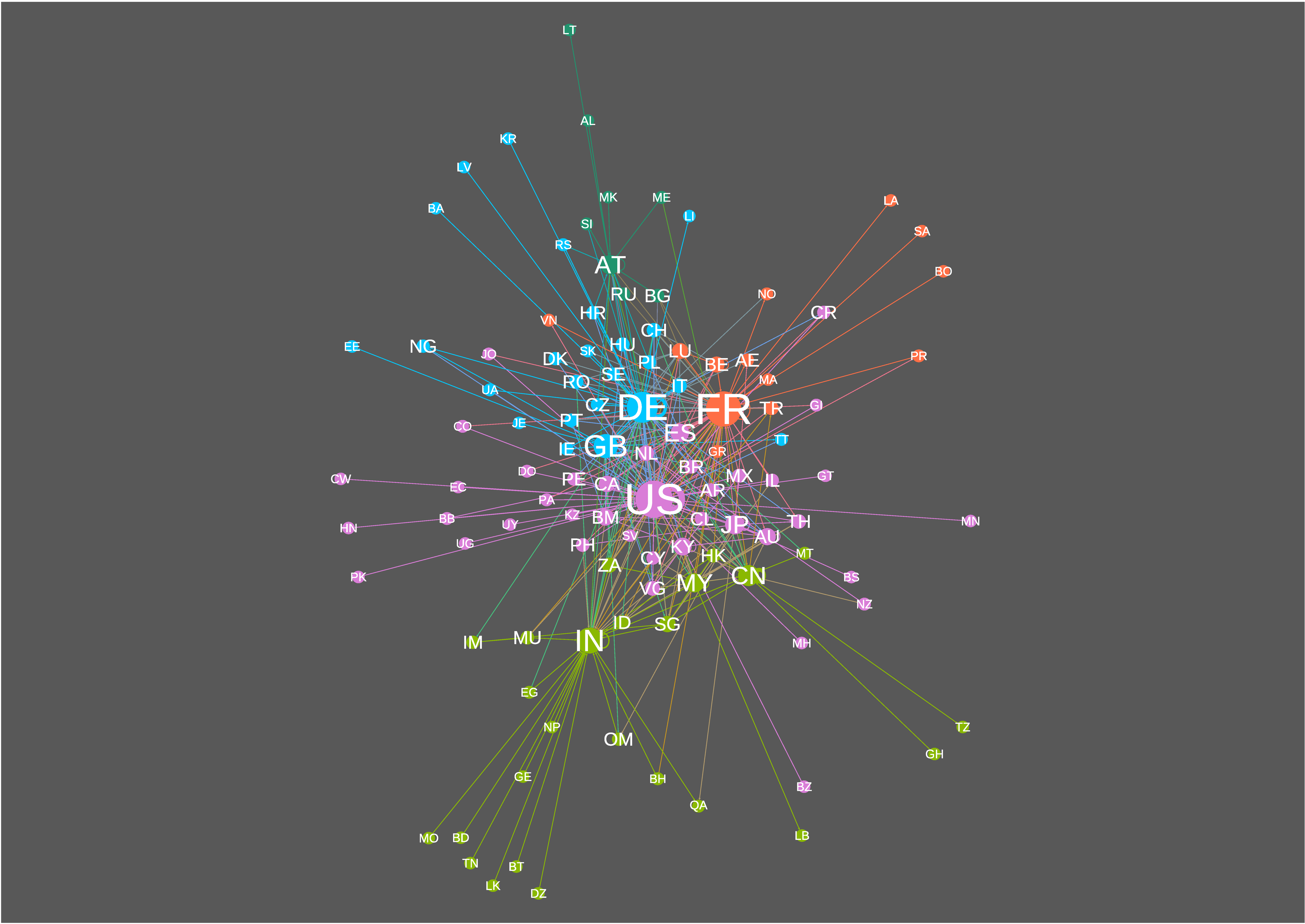

Supplement: S24 Fig — (TIF) [file pone.0255450.s024.tif]
